# Ion Conduction Mechanisms in Potassium Channels Revealed by Permeation Cycles

**DOI:** 10.1021/acs.jctc.3c00061

**Published:** 2023-04-11

**Authors:** Chun Kei Lam, Bert L. de Groot

**Affiliations:** Computational Biomolecular Dynamics Group, Max Planck Institute for Multidisciplinary Sciences, Göttingen 37077, Germany

## Abstract

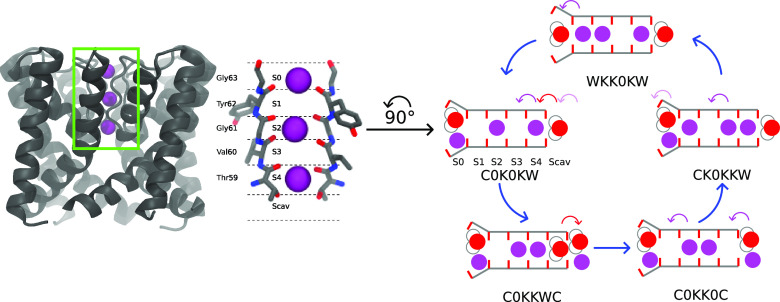

Potassium channels are responsible for the selective yet efficient permeation of potassium ions across cell membranes. Despite many available high-resolution structures of potassium channels, those conformations inform only on static information on the ion permeation processes. Here, we use molecular dynamics simulations and Markov state models to obtain dynamical details of ion permeation. The permeation cycles, expressed in terms of selectivity filter occupancy and representing ion permeation events, are illustrated. We show that the direct knock-on permeation represents the dominant permeation mechanism over a wide range of potassium concentrations, temperatures, and membrane voltages for the pore of MthK. Direct knock-on is also observed in other potassium channels with a highly conserved selectivity filter, demonstrating the robustness of the permeation mechanism. Lastly, we investigate the charge strength dependence of permeation cycles. Our results shed light on the underlying permeation details, which are valuable in studying conduction mechanisms in potassium channels.

## 1 Introduction

**Figure 1:**
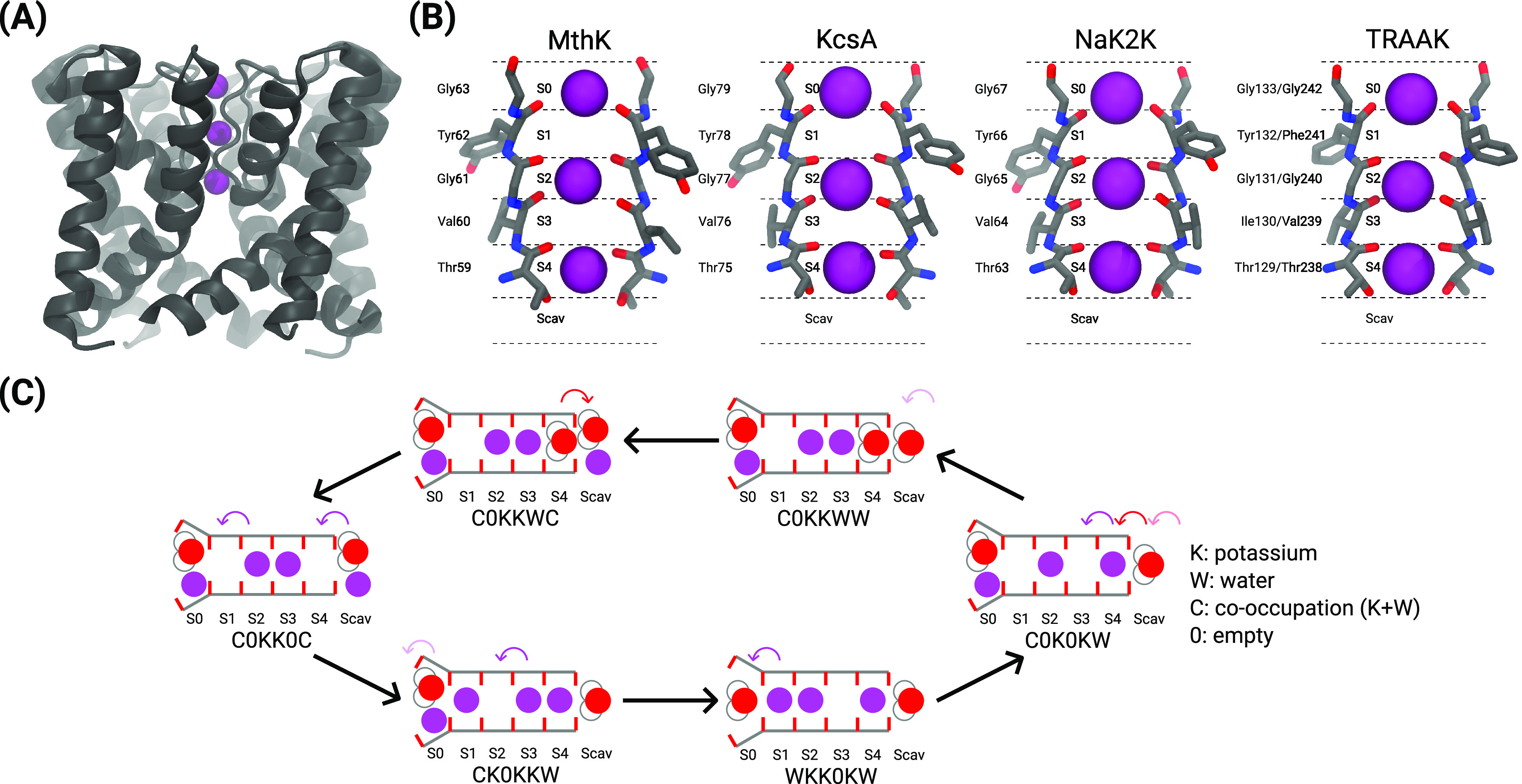
(A) Pore-only structure of MthK (PDB ID: 3LDC). (B) Selectivity filter (SF) of MthK, KcsA, NaK2K, and TRAAK. Each SF consists of six binding sites (S0 to Scav). Only two opposite subunits are shown for clarity. (C) Example of reduced permeation cycles.

Potassium (K^+^) channels are present in almost all organisms, mediating K^+^ fluxes during action potentials.^1^ Most K^+^ channels contain regulatory domain(s) and pore-forming domain(s), except for channels such as KcsA, which contains only the pore-forming domain.^2^ The regulatory domains are responsible for sensing stimuli and inducing conformational changes in the channel to regulate ionic fluxes through the pore. The pore-forming domains provide a direct passage of K^+^ across membrane bilayers (Figure 1A). Despite the structural variety of K^+^ channels due to variations in their pore-forming domains and regulatory domains, the selectivity filter (SF), a component of the pore domain central to ion permeation, is highly conserved. A signature sequence TVGYG constitutes a SF with 4-fold symmetry in K^+^ channels such as KcsA, MthK, and NaK2K. Substitutions of V by I and Y by F in pore domain 1 (P1) and pore domain 2 (P2), respectively, are observed in K2P channels such as TRAAK and TREK-2 and lead to a 2-fold symmetric SF. The SF forms the narrowest part of the channel and provides a conduction path to permeant ions. A canonical SF contains four main binding sites (S1, S2, S3, and S4) and two additional binding sites (S0 and Scav), which are exposed to the extracellular side and the cavity of the channel, respectively (Figure 1B). The binding sites provide coordination to K^+^, compensating for the loss of solvation upon entering the SF. S0 to S3 are formed by the backbone carbonyl oxygen atoms of the SF residues. S4 is formed by the backbone carbonyl oxygen atoms and side chain hydroxyl oxygen atoms from four threonines. Scav shares the side chain hydroxyl oxygen atoms with S4. It has been shown that the conduction properties of K^+^ channels are sensitive to the geometry of the SF. For instance, a study of the wild type (WT) and mutants of the NaK channel demonstrated that the channels with two or three binding sites lost their selectivity to K^+^ over Na^+^ and only when four binding sites were present were the selectivity and permeation rate restored.^3^ Structural plasticity is also needed to explain the efficient conduction of different ions in the nonselective channel NaK.^4^

Because of the conservation of the SF, K^+^ channels likely share the same or very similar permeation mechanisms. The debate about the permeation mechanisms in K^+^ channels has lasted for at least one decade. The first KcsA structure revealed a full electron density in all four binding sites of its SF. The SF occupancy was interpreted as a superposition of two configurations KWKW and WKWK (for S1 to S4), as it was believed that K^+^ in direct contact would be electrostatically unfavorable.^5,6^ This interpretation naturally gave rise to the soft knock-on hypothesis for ion permeation. In the soft knock-on permeation, ions and water molecules move concertedly through the SF, achieving an ion–water permeation ratio of 1:1.^7^ However, molecular dynamics (MD) simulations showed that an alternative permeation mechanism that involves no water copermeation is energetically possible.^8^ A study consisting of extensive MD simulations and crystallographic measurements challenged the traditional view of the ion permeation mechanism, suggesting that direct knock-on, where ion–ion head-on collisions in the absence of water inside the SF are the primary driving force of ion permeation, is the dominant permeation mechanism in K^+^ channels.^9^

Even with the availability of high-resolution structures of K^+^ channels, limited insights into the dynamical properties relevant to ion permeation can be gained from these static conformations. Advances in computational research enable us to extract detailed dynamical information about ion permeation at an atomistic scale to investigate the permeation mechanisms in K^+^ channels. To this end, we used MD simulations and Markov state models (MSMs) to examine how the occupancy of the SF evolves during ion permeation under different conditions, including K^+^ concentration, temperature, and membrane voltage. Additional simulations were carried out to identify variations in ion permeation patterns for different channels. Finally, we varied the charges of charged residues and ions to evaluate the dependence of the observed permeation patterns on the force field implementation. The MD trajectories, resulting in a total simulation time of 300 μs and thousands of K^+^ permeation events, provide extensive dynamical information from which the underlying permeation processes were studied.

## 2 Methods

### 2.1 MD Simulations

The MthK system was adopted from the work by Kopec et al.^10^ CHARMM-GUI^11−13^ was used to embed the pore-only structure of MthK (PDB: 3LDC(14)), KcsA E71A (PDB: 5VK6(15)), NaK2K F92A (PDB: 3OUF(3)), and TRAAK (PDB: 4I9W(16)) into a 1-palmitoyl-2-oleoyl-*sn*-glycero-3-phosphocholine (POPC) membrane bilayer. For NaK2K, the mutation F92A was performed using CHARMM-GUI. The systems were solvated and neutralized by KCl. Binding sites S1 to S4 of all channels were occupied by K^+^ in the starting structures for the MD simulations. Two force fields, Amber14sb^17^ and CHARMM36m,^18^ were used to model the systems. For Amber14sb, Berger lipids,^19,20^ the TIP3P water model,^21^ and Joung and Cheatham ion parameters^22^ were used. Aliphatic hydrogen atoms were replaced by virtual sites.^23^ Combined with the use of the LINCS algorithm,^24^ an integration time step of 4 fs was used for the Amber14sb simulations. A leapfrog algorithm was used as the integrator. The temperature was maintained using a velocity rescaling algorithm.^25^ The pressure was kept at 1 bar using a semi-isotropic Berendsen barostat.^26^ A cutoff of 1.0 nm was used for van der Waals interactions. The particle mesh Ewald (PME) algorithm^27^ with a 1.0 nm distance cutoff was chosen to compute electrostatic interactions. For CHARMM36m, CHARMM36 lipids,^28^ CHARMM TIP3P water model,^29^ and CHARMM ion parameters^30^ were used. The LINCS algorithm^24^ was used to constrain all bonds associated with hydrogen atoms. An integration time step of 2 fs was used for all CHARMM36m simulations. A cutoff of 1.2 nm was used for van der Waals forces, and the forces were switched smoothly to zero between 0.8 to 1.2 nm. The particle mesh Ewald (PME) method with a 1.2 nm distance cutoff was used for electrostatic interactions. The temperature was maintained using Nosé–Hoover thermostat.^31,32^ The pressure was kept at 1 bar using Parrinello–Rahman barostat.^33^ Simple harmonic distance restraints between the backbone oxygen atom of the *i*-th residue and the backbone hydrogen atom of the (*i* + 4)-th residue for residues between ACE17 (N-terminal acetyl capping group) and VAL30 and between PHE87 and NME100 (C-terminal *N*-methyl amide capping group) within the same chain were applied to avoid unfolding of the two termini in each monomer. The equilibrium distance *d*_0_ and the force constant *k* are 0.2 nm and 1000 kJ mol^–1^ nm^–2^, respectively. An external electric field *E* = *V*/*L*, where *V* is the membrane voltage and *L* is the length of the simulation box in the *z*-direction, was applied along the *z*-axis. For charge scaling, the scaling factor *q*/*q*_0_, where *q* is the scaled charge and *q*_0_ is the default charge of the charged residues of MthK, K^+^, and Cl^–^, was applied. In the case of charged residues, *q* was achieved by adding an offset charge of (*q* – *q*_0_)/*n*_nm_, where *n*_nm_ is the number of non-main chain atoms of the residue, to all the non-main chain atoms of the same residue. Also, as all partial charges were rounded to four decimal places, counter charges were added to C_β_ atoms to account for missing charges due to rounding to keep the simulation box neutral. All simulations were performed with GROMACS 2020.^34,35^ A summary of the simulations can be found in Tables S1, S2, S3, and S4.

### 2.2 Markov State Modeling and Permeation Cycles

The boundaries of the SF binding sites were defined by the z-coordinate of the center of mass (CoM) of the backbone carbonyl oxygen atoms or the hydroxyl oxygen atoms of residues of the SF. For instance, the upper boundary of S2 of MthK WT was defined by the z-coordinate of the CoM of the backbone carbonyl oxygen atoms from the four Gly61, as MthK is a tetramer. Similarly, the lower boundary of S2 was defined by the z-coordinate of the CoM of the backbone carbonyl oxygen atoms from the four Val60. In Figure 1B, S2 is occupied by a K^+^, as a K^+^ is found between the two boundaries of S2 and within 4 Å of the axis of symmetry of the SF. The upper boundary of Scav was formed by the hydroxyl oxygen atoms of four threonine residues, and the lower boundary of Scav was set to be 4 Å below its upper boundary. Using these definitions, we expressed the SF occupancy of each simulation snapshot using a six-character code, with each character representing the occupancy of one binding site. For the state C0K0KW in Figure 1C, since S0 is occupied by a potassium ion and a water molecule at the same time, C (Co-occupation) is assigned to the first letter. The second letter is 0, as S1 is vacant. Letters K and W represent the occupation by a potassium ion and a water molecule, respectively. The resulting six-character code defines a SF occupation state.

The transition matrix ***T***(τ) of a MSM using the SF occupation states was obtained via normalizing the count matrix ***C***(τ) by
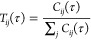
1where *T*_*ij*_(τ) is the probability of transitioning from state *S*_*i*_ to state *S*_*j*_ and *C*_*ij*_(τ) is the number of transitions from state *S*_*i*_ to state *S*_*j*_ after a lag time τ observed in MD simulations.

Performing eigendecomposition on ***T*** yields eigenvalues λ_*m*_ and eigenvectors **ν**_*m*_ that characterize the dynamical processes in the molecular system on different time scales.^36^ The first eigenvalue λ_1_ has a value of 1, corresponding to the steady-state distribution of the SF occupation states. Since the system exhibited nonequilibrium dynamics, λ_*m*_ and **ν**_*m*_ could be complex-valued for *m* > 1. We took the norm of λ_*m*_ when computing the *m*-th relaxation time *t*_*m*_, given by
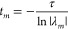
2*t*_*m*_ as a function of τ is plotted in Figure S2.

Another test checking whether Markov properties hold is the Chapman–Kolmogorov test,^36−38^ justifying to what extent the approximation
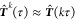
3for *k* = 2, 3, ... is fulfilled. τ was chosen to be 20 ps. Details can be found in the Supporting Information.

Independent permeation events, each capturing a series of transitions between SF occupation states that results in a K^+^ arriving at the extracellular side while the SF returns to its original occupation state, were first extracted from the trajectories using Algorithm 1. It assumes that a positive membrane voltage is applied. WKK0KW was selected to be the initial and the final state (denoted as *S*_*c*_) of the cycles as it was one of the states with a high probability of being observed under different simulation conditions. For instance, 98% and 100% of the observed permeation events in Figure 3 can be expressed in cycles that start and end in WKK0KW for Amber14sb and CHARMM36m, respectively. Using Algorithm 2, each of these permeation cycles was reduced such that only the nonrepeating, first-arrived states remain. Trivial oscillations between SF occupation states that involve no net ion jumps were removed. From the reduced permeation cycle trajectories, transition matrices, containing the probabilities of observing a transition between any two SF occupation states in a reduced permeation cycle, were computed. Apart from transition probabilities based on the reduced permeation cycles, the net fluxes *f*_*ij*_ = *T*_*ij*_ρ_*i*_ – *T*_*ji*_ρ_*j*_, where ρ_*i*_ and ρ_*j*_ are the steady-state distribution of states *S*_*i*_ and *S*_*j*_, respectively, between *S*_*i*_ and *S*_*j*_ were computed from the full trajectories without cycle identification and reduction. The currents through the channels were calculated as , where *J*_*k*_ is the total number of net ion jumps in the SF throughout a simulation (see Figure S1 for details of ion jumps in the SF).

The mean first passage time (MFPT), for the transitions from state *S*_*i*_ to state *S*_*j*_, is
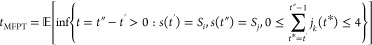
4where *s*(*t*) is the SF occupation state and *j*_*k*_(*t*) is the number of ion jumps at time *t* (see Figure S1), was computed. The last condition restricts the calculations to transitions within the same permeation cycle.

The permeation cycle analysis was performed using KPerm, a Python package developed by us. Libraries including MDAnalysis,^39,40^NumPy,^41^ and SciPy(42) were used to process and analyze simulation trajectories. NetworkX(43) was used to generate graphs of permeation cycles. Errors of MFPTs are bias-corrected and accelerated (BCa) 95% bootstrap intervals (*B* = 10000). Unless otherwise specified, errors are 95% confidence intervals based on the *t*-distribution.

## 3 Results

### 3.1 Selectivity Filter Occupancy

To probe the occupancy of SF during ion permeation, we carried out MD simulations of the pore of MthK WT, KcsA E71A, NaK2K F92A, and TRAAK WT in a 1 M KCl solution at 323 K and 300 mV. Mutants of KcsA^44^ and NaK2K^45^ were chosen to obtain more permeation events as they have been shown to have higher conductance than WT. Since hundreds of K^+^ permeation events in total for each system were recorded from independent simulations (Tables S1, S2, S3, and S4), the average occupancy is not merely a consequence of the initially full K^+^ occupancy of the SF but the preference of permeant ions and water molecules for SF binding sites during ion permeation.

**Figure 2:**
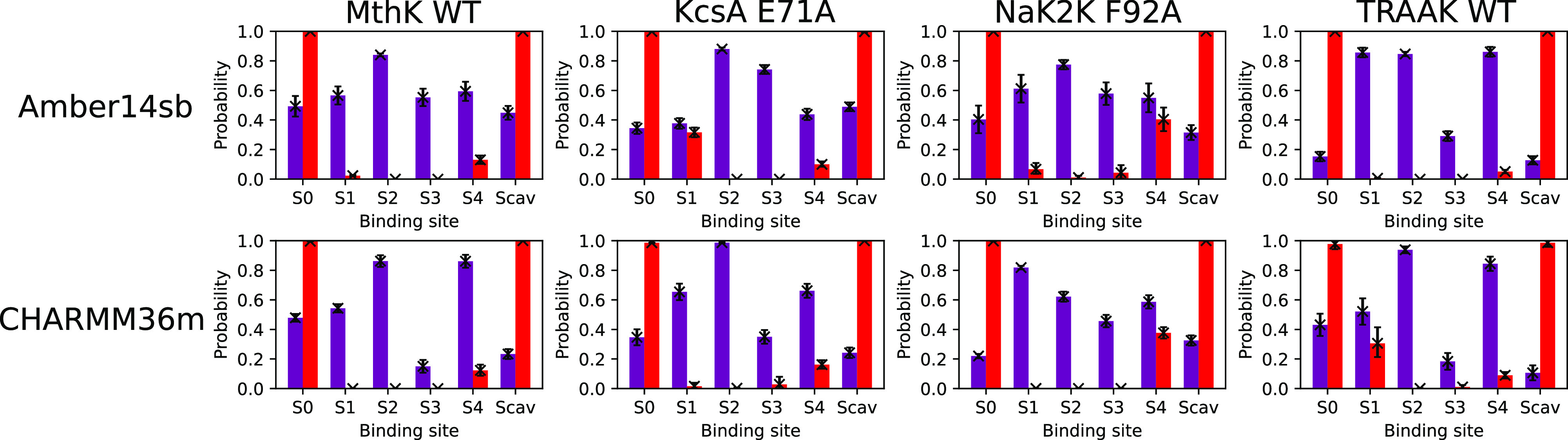
K^+^ (purple) and water (red) occupancy in SF binding sites of channels simulated at 323 K and 300 mV in 1 M KCl solution. Errors are 95% confidence intervals based on the t-distribution, with the number of observations equal to the number of independent trajectories.

S0 and Scav are exposed to water and thus have a nearly full water occupancy throughout the simulations of all channels (Figure 2). The inner binding sites S2 and S3 are mostly shielded from water. In MthK, S2 and S3 are completely dehydrated in simulations using Amber14sb and CHARMM36m. Nonzero water occupancy in S2 and S3, caused by rare events of water entering S2 or S3 from the cytosolic side in a few trajectories, is found in other channels. Water molecules hopping between S0 and S1 and between S4 and Scav happens, contributing to the water occupancy in S1 and S4, respectively. The high K^+^ density in S1 to S4 and the dehydration of S2 and S3 suggest that the K^+^ permeation happens predominately without water copermeation. However, how water molecules contribute to the water-free K^+^ permeation and under what conditions water copermeation is triggered are not fully understood.

There are variations in K^+^ and water occupancy between the channels with a highly conserved SF. The average occupancy of K^+^ and water is also sensitive to the choice of force fields. For instance, the K^+^ occupancy of S3 in CHARMM36m simulations tends to be substantially lower than that in Amber14sb simulations. S1 water occupancy of ∼30% is found in simulations of KcsA E71A using Amber14sb and simulations of TRAAK WT using CHARMM36m but not in simulations using their counterpart force fields. The presence of water in the SF binding sites and variations in SF occupancy due to force fields and structural differences between channels prompt the questions about permeation mechanisms in these systems and call for a dissection of the permeation processes in the SF.

### 3.2 Permeation Cycles

In search of the dynamical details of permeation processes in the SF of K^+^ channels, we turned to Markov state modeling and expressed ion permeation events during the MD simulations as transitions between SF occupation states. The resulting MSMs contain the arrays of six-character code that represent the occupancy of the six binding sites. Reduced permeation cycles were obtained by isolating permeation events from the trajectories and removing trivial oscillations between the states that do not result in net ion movement. Figure 1C shows an example of reduced permeation cycles. A closed loop in a permeation cycle depicts the sequence of SF occupation states for a permeation event in which the SF occupancy is restored to its initial configuration, thus completing a permeation event. With that, one K^+^ has reached the extracellular side of the membrane through the SF.

**Figure 3:**
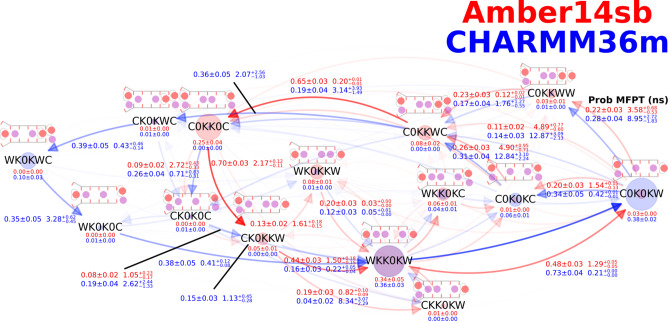
Permeation cycles and MFPTs of SF occupation state transitions for MthK WT at 300 mV and 323 K in 1 M KCl solution. Only states involved in more than 10% of the observed permeation cycles are shown. The steady-state distributions of the SF occupation states are stated below the nodes. The probabilities of observing the transitions given a reduced permeation cycle and the associated MFPTs are stated next to the edges. Cycles of 98% and 100% of the permeation events for Amber14sb and CHARMM36m, respectively, were identified and are shown.

The currents in MthK WT, with the simulated conditions, are 15.4 ± 2.2 pA and 6.8 ± 2.0 pA (errors representing 95% confidence intervals using the t-distribution computed from 20 independent simulations) for Amber14sb and CHARMM36m, respectively. The reduced permeation cycles for MthK WT are displayed in Figure 3. The state WKK0KW was chosen to be the initial and final state of the cycles as it is one of the states with the highest probability under different simulation settings, including channels, force fields, K^+^ concentration, temperature, and membrane voltage. For instance, 98% and 100% of the permeation events can be expressed as cycles starting and ending in WKK0KW for MthK WT using Amber14sb and CHARMM36m, respectively, at 300 mV and 323 K in a 1 M KCl solution. The permeation cycles are consistent with the plots using net fluxes as the weights of the edges (Figure S9).

Starting in WKK0KW, as the permeation proceeds, the first transition is predominantly to C0K0KW for both force fields. The transition represents the K^+^ in S1 moving to S0 and sharing the same binding site with water. It was found in 48 ± 3% and 73 ± 4% of the reduced permeation cycles for Amber14sb and CHARMM36m, respectively (note that these probabilities are different from the transition probabilities in the MSMs). Three routes, ultimately converging to C0KKWC, branch out of C0K0KW. Two steps in common are involved: a K^+^ and a water molecule in S4 and Scav hopping collectively to S3 and S4 at the same time, respectively, and a K^+^ approaching from the channel cavity to Scav. One of the three routes is the direct transition C0K0KW → C0KKWC, in which the two steps happen simultaneously for a lag time of 20 ps. The other two routes, which occur approximately twice as frequently as the direct transition, differ in the order of the two steps. C0K0KW → C0K0KC → C0KKWC represents the formation of a central ion pair in S2 and S3 with the presence of a K^+^ in Scav. The last route C0K0KW → C0KKWW → C0KKWC suggests the possibility of spontaneous formation of a central ion pair without head-on collisions by a third K^+^ in Scav.

Critical differences in permeation cycles between Amber14sb and CHARMM36m emerge at C0KKWC. For Amber14sb, the cycles continue with the depletion of a transient water molecule in S4 (C0KKWC → C0KK0C, 65 ± 3%), followed by a concerted forward movement of two ions, moving from S2 to S1 and Scav to S4 (C0KK0C → CK0KKW, 70 ± 3%), respectively. The dominant cycle is eventually closed by the dissociation of K^+^ in S0 and the forward movement of K^+^ from S3 to S2. The majority of the permeation events for Amber14sb can be represented by a single loop that splits at CK0KKW and converges at C0KKWC. In contrast, a divergence into two distinct permeation routes at C0KKWC was observed for CHARMM36m. One of the two routes is similar to the Amber14sb permeation route, involving water depletion in S4, except that CK0K0C plays a more significant role in connecting C0KK0C and WKK0KW. Compared to the first permeation route, the depletion of transient water in S4 happens later in the second route. Ion movement facilitated by ion–ion repulsion (C0KKWC → CK0KWC → WK0KWC) finishes earlier than water depletion (WK0KWC → WK0K0C). The cycle ends with the K^+^ coming from Scav to S4 and the K^+^ in S3 moving to S2 (WK0K0C → WKK0KW).

Mean first passage times (MFPTs) of the transitions were computed. The permeation cycles are comprised of transitions on a broad range of time scales between tens of picoseconds and tens of nanoseconds. For both force fields, the rate-limiting step is the formation of a central ion pair in S2 and S3 with vacant S1 (C0KKxx), likely due to the barrier posed by the strong Coulomb repulsion between the two K^+^s. Consistent with the lower current in CHARMM36m simulations, most rate-limiting permeation steps are considerably slower in CHARMM36m simulations than in Amber14sb simulations. All the slowest steps in the three transition routes from C0K0KW to C0KKWC for CHARMM36m are about 2.5 times as slow as for Amber14sb. No permeation cycle finishes substantially faster than the others, suggesting that there is unlikely a permeation “short-cut” at a significantly higher rate that the channel may exploit to regulate ion conduction rate by switching between different permeation routes.

The permeation cycles in MthK represent the direct knock-on permeation, characterized by the close K^+^ contact and the absence of water in S2 and S3. Despite the presence of water in S1 and S4, no water permeation event was observed. The water occupancy in S1 does not contribute to K^+^ permeation, as no relevant SF occupation state is found in the permeation cycles. The transient S4 water occupancy shown in Figure 2 can be explained by the high probability of finding states with a water molecule in S4, including C0KKWC and WK0KWC, in a reduced permeation cycle. C0KKWC is achieved by a water molecule entering the SF during the formation of a central ion pair. The escorting water molecule enters the channel simultaneously with a K^+^ jumping from S4 to S3. It appears to facilitate the formation of a central ion pair essential for the subsequent ion permeation steps. After forming the central ion pair, the water molecule escapes from S4 to Scav in spite of the presence of K^+^ in the adjacent binding sites S3 and Scav. This lingering water molecule may be due to the high cost of complete dehydration of K^+^ when traversing through the SF.

The permeation patterns are sensitive to the choice of force fields. With the identified permeation cycles, we can explain the lower K^+^ occupancy, compared to Amber14sb, in S3 for CHARMM36m. There is a higher probability of finding states such as C0KK0C, C0KKWC, CK0KKW, or WK0KKW, which has a K^+^ in S3, in Amber14sb simulations then in CHARMM36m simulations. The permeation routes sampled by the two force fields manifest the direct knock-on mechanism, yet variations in the details of the permeation cycles exist.

### 3.3 Diversity of Permeation Cycles

**Figure 4:**
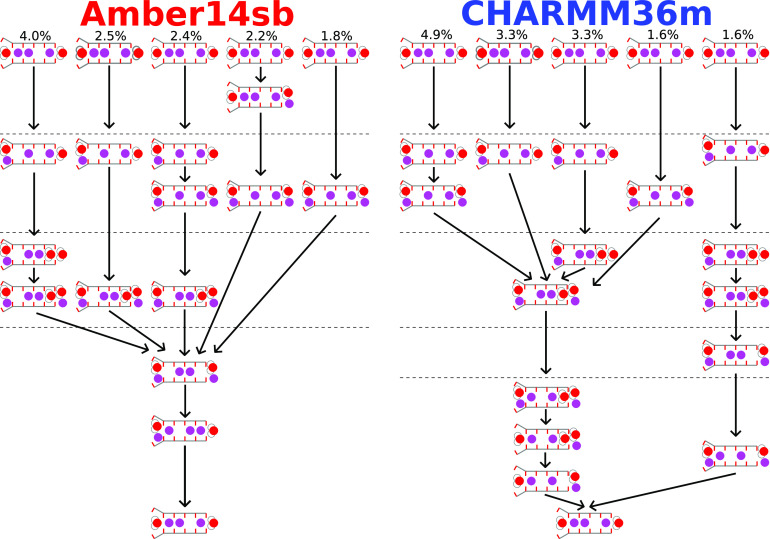
Diversity of permeation cycles. The first five most frequently observed permeation cycles are shown.

We examined the permeation cycles individually. As shown in Figure 4, the most frequently observed permeation cycles account for only 4.0% and 4.9% of the total observed cycles in Amber14sb and CHARMM36m simulations, respectively. Upon closer examination of the permeation cycles, the apparently highly diversified permeation cycles exhibit many common features. For instance, the cycles converge to the same route as the permeation proceeds. The observed cycles share multiple intermediate SF states that emerge in the same order in time to connect the cycles. While there are numerous nonidentical permeation cycles, the ion permeation is not about random occupancy of the SF but collective motions of K^+^ and water with patterns. The observed permeation cycles carry a lot of common features that one can extract to analyze the underlying conduction mechanisms in the simulations.

### 3.4 Effects of Potassium Concentration

The permeation cycle analysis allows a systematic investigation into the effects of physical factors, such as K^+^ concentration, temperature, and membrane voltage, on the permeation mechanisms in K^+^ channels. While it is expected that increasing K^+^ concentration leads to increased ionic current, microscopic details of the impacts of K^+^ concentration on ion conduction remain elusive.

**Figure 5:**
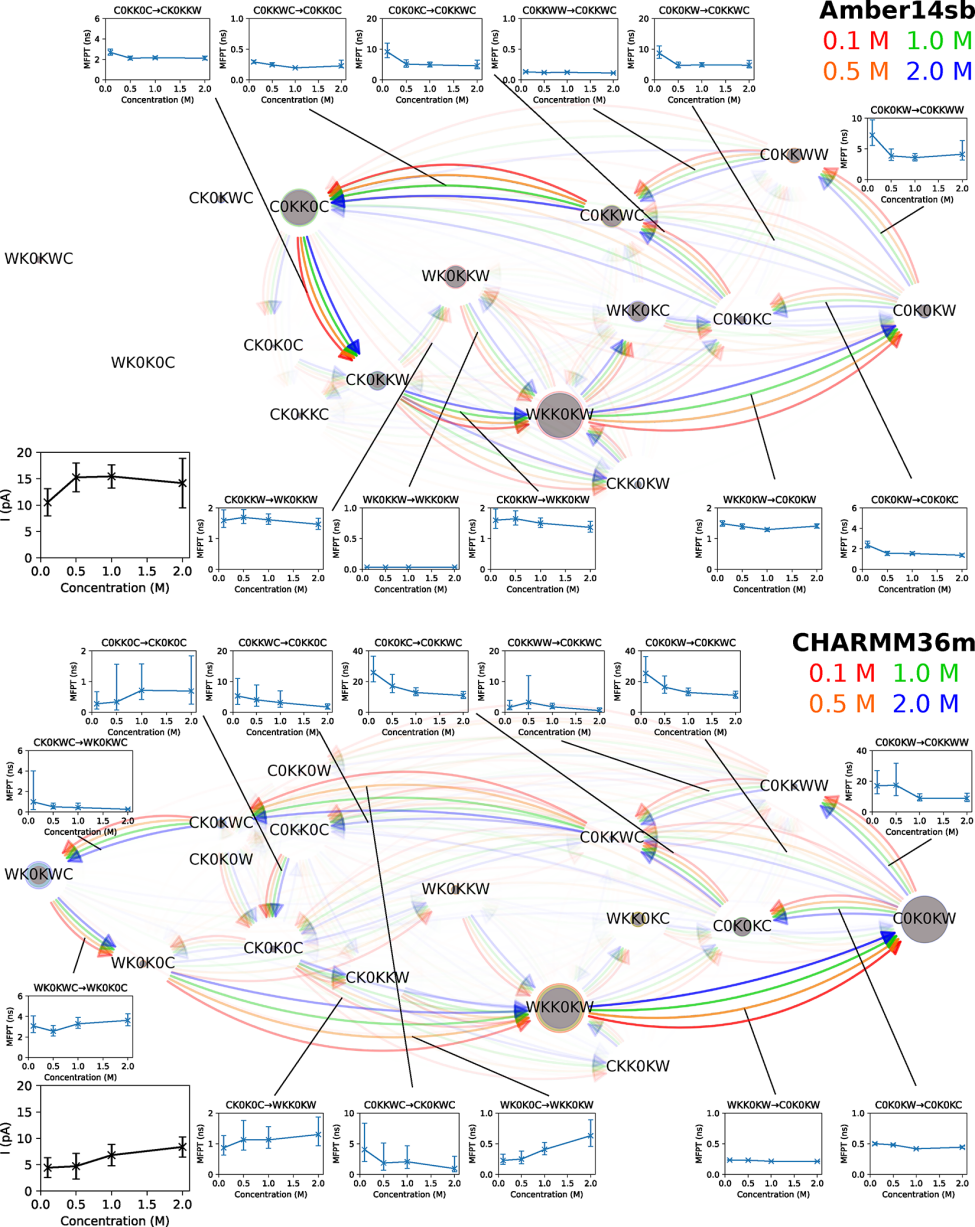
Permeation cycles, MFPTs of SF occupation state transitions, and currents for MthK at 300 mV and 323 K in KCl of different concentrations. See the description in Figure 3 for the meaning of nodes and edges. Insets are the currents and the MFPTs of the transitions as a function of KCl concentration. Cycles of 98%, 97%, 98%, and 97% for Amber14sb and 99%, 100%, 100%, and 96% for CHARMM36m of the permeation events in a 0.1, 0.5, 1.0, 2.0 M KCl solution, respectively, were identified and are shown.

We, therefore, carried out simulations of MthK WT in a KCl solution of concentrations ranging from 0.1 to 2.0 M and analyzed the permeation cycles (Figure 5). The channel remains conductive in all simulations. No significant conformational change of the SF was observed, even for simulations which show low K^+^ permeation counts, suggesting that the pore domain of MthK is stable over a wide range of KCl concentrations for at least 500 ns. Surprisingly, even with a 20-fold increase in KCl concentration, many details of the permeation, including the preferred transition paths and the steady-state probabilities of SF occupation states, remain largely unaffected. The acceleration in ion permeation is mainly attributed to the shorter MFPTs of the rate-limiting steps, approximately reduced by half when increasing the K^+^ concentration from 0.1 to 2.0 M. Although the two force fields sample different permeation routes, similar effects of salt concentration on ion permeation were observed in Amber14sb and CHARMM36m simulations.

Interestingly, one water permeation event happens in one of the CHARMM36m simulations with a K^+^ concentration of 2.0 M. A water molecule stalls the channel for ∼250 ns until it has crossed the SF. Afterward, the channel resumes the water-free direct knock-on permeation and remains conductive to K^+^ for the rest of the simulation. Therefore, water permeation is possible yet extremely rare under the given simulation conditions.

### 3.5 Effects of Temperature

**Figure 6:**
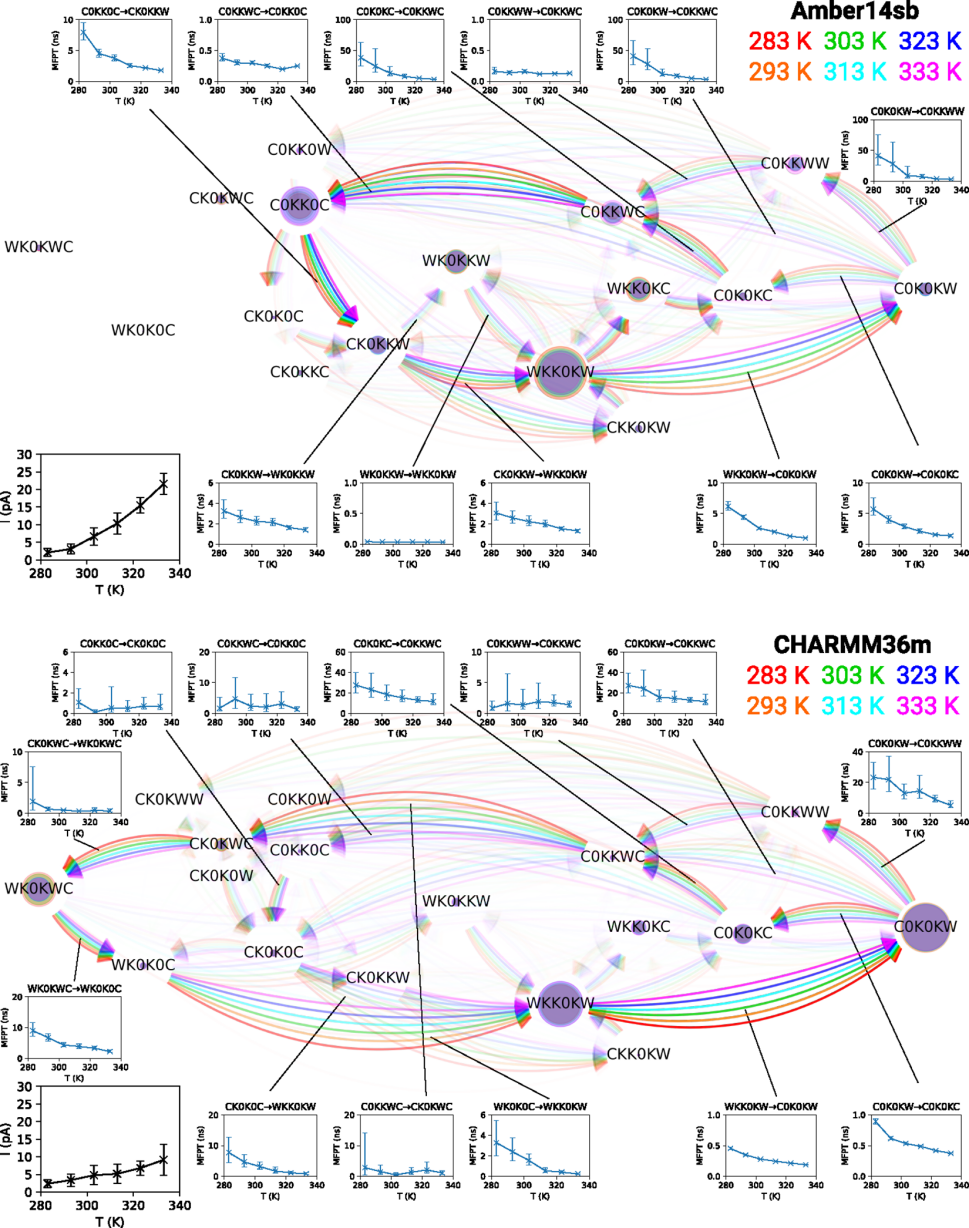
Permeation cycles, MFPTs of SF occupation state transitions, and currents for MthK at 300 mV and different temperatures in 1 M KCl. See the description in Figure 3 for the meaning of nodes and edges. Insets are the currents and the MFPTs of the transitions as a function of temperature. Cycles of 97%, 98%, 97%, 98%, 98%, and 98% for Amber14sb and 100%, 100%, 100%, 100%, 100%, and 100% for CHARMM36m of the permeation events at 283, 293, 303, 313, 323, and 333 K, respectively, were identified and are shown.

The temperature dependence of permeation cycles in MthK was also probed. As shown in Figure 6, similar to the K^+^ concentration dependence, the permeation patterns exhibited by the channel are largely invariant even when elevating the temperature drastically from 283 to 333 K (well above 270 K, the phase transition temperature of pure POPC bilayers^46^). While increasing the K^+^ concentration primarily speeds up the rate-limiting steps of ion conduction, the higher temperature accelerates most SF occupation state transitions relevant to permeation. Although the ion conduction rate increases substantially at higher temperatures, no water copermeation was observed. We conclude that the permeation mechanism of the pore domain of MthK is insensitive to temperatures between 283 and 333 K.

The free energy differences between SF occupation states of KcsA were found to be insensitive to the temperature for CHARMM36m.^47^ Given the structural similarities between the SF of KcsA and MthK, we believe that the conductance of MthK increases with temperature primarily due to more frequent attempts of ions overcoming the barriers during all permeation steps.

### 3.6 Effects of Membrane Voltage

**Figure 7:**
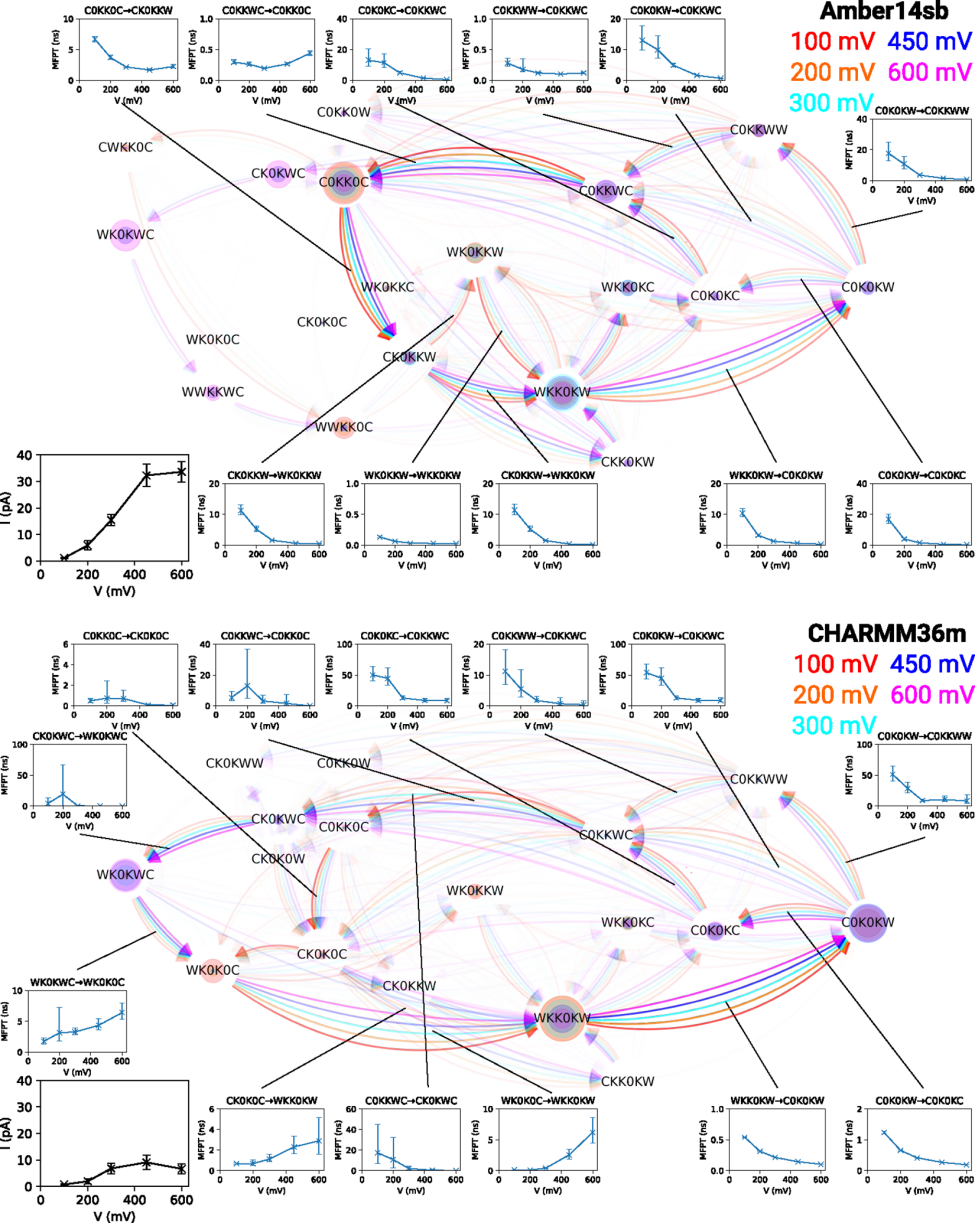
Permeation cycles, MFPTs of SF occupation state transitions, and currents for MthK at different membrane voltages and 323 K in 1 M KCl. See the description in Figure 3 for the meaning of nodes and edges. Insets are the currents and the MFPTs of the transitions as a function of voltage. Cycles of 88%, 98%, 98%, 96%, and 90% for Amber14sb and 100%, 100%, 100%, 95%, and 87% for CHARMM36m of the permeation events at 100, 200, 300, 450, and 600 mV, respectively, were identified and are shown.

Membrane voltage influences the rates of most of the permeation steps (Figure 7). As the membrane voltage increases, transition pathways and SF occupation states are redistributed, more significant than what was identified when varying K^+^ concentration or temperature. While the permeation patterns are moderately conserved at a membrane voltage below 300 mV, there is a shift in permeation pathways toward the alternative route C0KKWC → CK0KWC → WK0KWC for both force fields at high voltages (450 mV and 600 mV). In CHARMM36m simulations, a high voltage promotes this route which is present under most of the conditions we explored. In Amber14sb simulations, this alternative route emerges only at high voltages. The route is connected with states WWKKWC and WWKK0C, which have high water content and are irrelevant to permeation under most of the explored conditions. The permeation at high voltages is more chaotic, as only 96% and 90% for Amber14sb and 95% and 87% for CHARMM36m of the permeation events can be described in closed cycles that start and end in WKK0KW at 450 mV or 600 mV, respectively. We observed no water permeation event at physiologically relevant voltages (50 mV and 100 mV, see Tables S1 and S3). A few water copermeation events were identified at high voltages. The tendency of water traversing the SF is mechanistically similar to the so-called water finger protrusion that happens before pore formation induced by a strong electric field in lipid bilayers.^48^ Since the voltage drop happens mainly in the SF, the electric field  along the channel axis inside the SF increases with the applied membrane voltage. The force exerted on a dipole increases with the increase in electric field strength inside the SF. The strengthened coupling of dipoles to the electric field drives water into the SF.^49^ The current not increasing with the voltage at a high limit is likely due to the chaotic permeation triggered by the propensity of water copermeation at an exceedingly high voltage, which slows down the ion permeation.

### 3.7 Permeation Cycles in Different Channels

**Figure 8:**
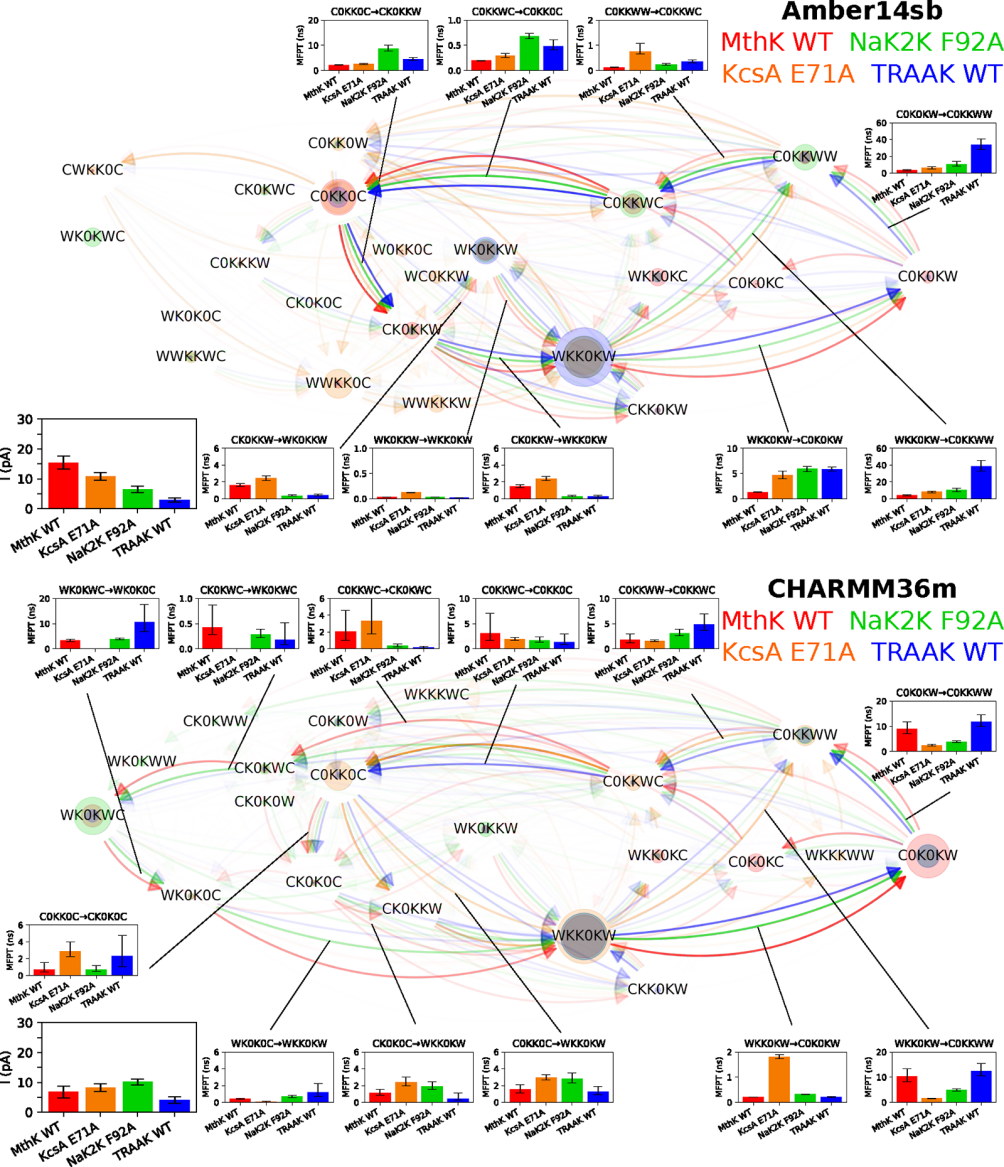
Permeation cycles, MFPTs of SF occupation state transitions, and currents for MthK WT, KcsA E71A, NaK2K F92A, and TRAAK at 300 mV and 323 K in 1 M KCl solution. See the description in Figure 3 for the meaning of nodes and edges. Insets are the currents and the MFPTs of the transitions. Cycles of 95%, 93%, 91%, and 86% for Amber14sb and 94%, 89%, 95%, and 83% for CHARMM36m of the permeation events in MthK WT, KcsA E71A, NaK2K F92A, and TRAAK, respectively, were identified and are shown.

Next, we explored how the permeation mechanisms differ for different channels with a SF with an identical or slightly different amino acid sequence. The observed currents for MthK WT, KcsA E71A, NaK2K F92A, and TRAAK WT are different (Figure 8). Noticeable differences in the steady-state distribution and preferred transitions between SF occupation states during ion permeation were also found. Starting from WKK0KW, C0K0KW is usually visited before C0KKWW for MthK WT. In contrast, KcsA E71A, NaK2K F92A, and TRAAK WT can skip C0K0KW, transitioning from WKK0KW to C0KKWW directly in one step for a lag time of 20 ps. Another deviation from MthK WT is that all other channels prefer C0K0KW → C0KKWW → C0KKWC to C0K0KW → C0K0KC → C0KKWC, but these two paths are equally populated in MthK WT. The path C0KKWC → CK0KWC → WK0KWC → WK0K0C → WKK0KW present in CHARMM36m simulations is prevalent in MthK WT and NaK2K F92A, but it is almost absent in KcsA E71A and TRAAK WT. In Amber14sb simulations, KcsA E71A uses an alternative path C0KK0C → CWKK0C → WWKK0C → WWKKKW → WKK0KW for K^+^ permeation.

Along with hundreds of K^+^ permeation events, a few water permeation events were identified in simulations of KcsA E71A and TRAAK WT using CHARMM36m. This observation reveals again that water permeation is a possible yet rare event. Different channels may have different voltage tolerances as an exceedingly high membrane voltage likely triggers water copermeation. Most observed permeation events involve no water permeation, implying the dominance of the water-free direct knock-on mechanism in K^+^ channels. The formation of the central ion pair remains the slowest permeation step for all channels, except for KcsA E71A simulated with CHARMM36m, as several slowest permeation steps have similar MFPTs. We conclude that there are channel-specific variations in permeation cycles among the channels, but the direct knock-on permeation mechanism and most permeation patterns remain highly conserved.

### 3.8 Charge Strength Dependence of Permeation Cycles

**Figure 9:**
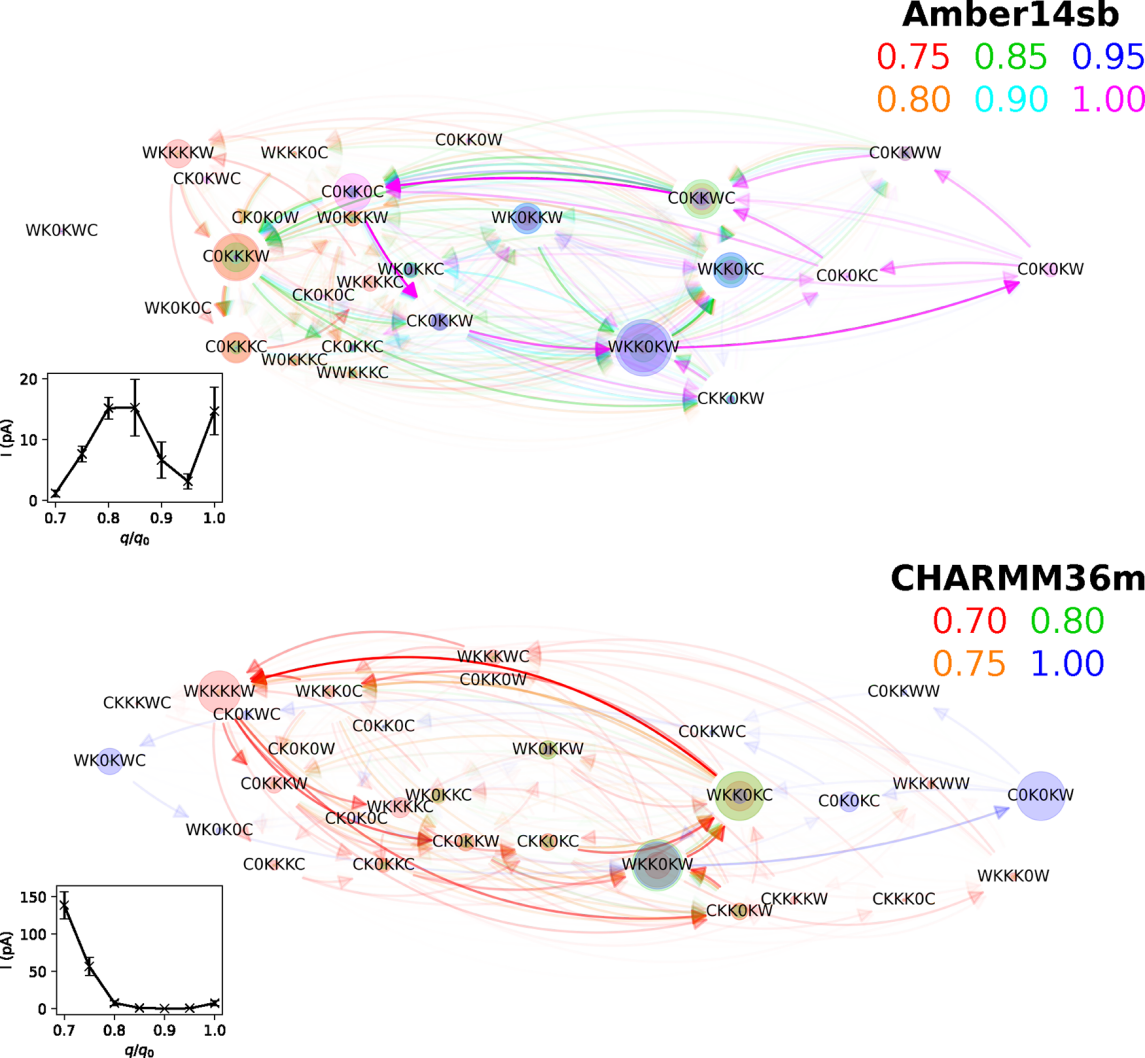
Net fluxes between SF occupation states for MthK at 0.035 V nm^–1^ and 323 K in 1 M KCl with different charge scaling factors *q*/*q*_0_. Node sizes scale with the steady-state distributions of the SF occupation states. Edges represent the net fluxes between states. Only the net fluxes larger than 0.15 and 0.03 of the maximum among all net fluxes are shown for Amber14sb and CHARMM36m, respectively.

Modifying intermolecular interaction parameters of a force field to reproduce realistic behaviors of biological systems is a popular technique for force field optimization.^50,51^ One possible way is to scale the charges of charged molecular groups, such as charged residues and ions.^52^ Since we have demonstrated that different permeation patterns arise from Amber14sb and CHARMM36m, scaling the charges is expected to alter the conductance and permeation patterns of the channels. To explore the charge strength dependence of permeation cycles, we performed simulations of MthK WT with different charge scaling factors *q*/*q*_0_ for charged residues and ions. The external electric field strength was kept at 0.035 V nm^–1^, equivalent to 300 mV for the unmodified system. The calculated currents were based on the number of ion jumps *j*_*k*_ through the SF and not corrected for the scaled charges. Using Amber14sb, the conductance of MthK fluctuates when *q*/*q*_0_ decreases from 1.00 to 0.70. For CHARMM36m, the conductance drops from 6.81 ± 2.01 pA to 0.32 ± 0.24 pA when *q*/*q*_0_ decreases from 1.00 to 0.90 and rises drastically to 138.9 ± 18.1 pA when *q*/*q*_0_ decreases to 0.70 (Figure 9). There are substantial shifts in the permeation patterns as the charges are reduced, causing the state WKK0KW to no longer be visited as frequently, especially when the factor is small (≤0.75). As a result, most of the permeation events cannot be represented as cycles that start and end in WKK0KW. Instead, the net fluxes in different systems are compared. For both force fields, routes involving a high number of ions inside the SF dominate the permeation cycles when the charges are scaled down, consistent with the intuition that the reduced charges lead to weakened electrostatic repulsion between ions and favor more ions occupying the binding sites simultaneously.

## 4 Discussion

### 4.1 Conduction Properties under Different Conditions

Unlike KcsA, which has the same signature sequence TVGYG as MthK for the SF, X-ray crystallography revealed that the SF of MthK remained conductive and structurally unchanged in K^+^ concentration between 6 mM and 150 mM of K^+^ and suggested that K^+^ concentration below 1 mM was needed to deplete most ions in the binding sites and induce the collapse of the SF.^53^ MD simulations showed that the SF of MthK could collapse in 0 mM K^+^ due to the absence of K^+^ in S2. However, introducing one K^+^ in either S1, S2, or S3 prevented SF from collapsing and helped maintain a conductive conformation.^53^ Due to the small size of the simulation box used in our study, we could not decrease the KCl concentration further to probe the ion permeation and the conformational changes of MthK WT at a K^+^ concentration lower than 0.1 M while keeping a reasonable number of mobile ions for neutralizing the system and maintaining ionic fluxes through the channel. Our MD simulations demonstrate that the pore of MthK allows continuous K^+^ permeation in a KCl solution at a concentration as low as 0.1 M and as high as 2.0 M.

Single-channel recordings of MthK revealed a high temperature sensitivity between 294 and 312 K. N-terminal deletion constructs of MthK in the presence of 0.1 mM calcium showed a 20-fold increase in open probability (*P*_*o*_) by increasing the temperature.^54^ The temperature dependence was found to originate from the coupling between the pore domain and the RCK domain. As the temperature increased, the coupling appeared to be disrupted. The same temperature dependence was not observed from the pore-only structure of MthK, a truncated construct in which the N-terminal segment and the RCK domain were absent, similar to the structure we simulated. At −100 mV, increasing the temperature from 293 to 309 K led to an approximately 4-fold decrease in *P*_*o*_ while the unitary conductance increased slightly. In agreement with the experimental observation of increased single-channel conductance, the pore domain of MthK displays higher currents at +300 mV upon elevated temperature over the range between 283 to 333 K in our MD simulations.

Even though MthK lacks a canonical voltage-sensing domain,^55^ it possesses a voltage-dependent gate reminiscent of the C-type inactivation gate located at the selectivity filter.^56^ At negative potentials, *P*_*o*_ remains high and increases slightly with voltage. Upon depolarization, a drastic reduction in *P*_*o*_ with voltage was observed. No event of channel closing was observed from our simulations. Since increasing external K^+^ concentration increases *P*_*o*_ at positive potentials and stabilizes MthK in the open state,^57^ observing no significant conformational change of the selectivity filter at a high voltage that characterizes a transition from the open state to the closed state during our simulations could result from the stabilization by the high K^+^ concentration. In qualitative agreement with the single-channel recording,^57,58^ the simulated currents in MthK WT channels increase with the magnitude of the applied voltage between +100 mV and +200 mV.

### 4.2 Robustness of Direct Knock-on

Consensus on whether soft knock-on or direct knock-on is the dominant permeation mechanism used by most K^+^ channels is, as of now, not fully reached.^59^ Markov state modeling allows for an in-depth analysis of the conduction events and the associated mechanisms in K^+^ channels.^60,61^ Here, for both Amber14sb and CHARMM36m, most permeation events observed in our simulations of MthK WT over a wide range of physical conditions, including K^+^ concentration, temperature, and membrane voltage, happen via direct knock-on. Direct knock-on is also the major permeation mechanism in KcsA E71A, NaK2K F92A, and TRAAK WT, all of which share a largely conserved SF with MthK WT. Permeation events can be represented by cycles of transitions between SF occupation states. The identified cycles are apparently diversified yet have many features in common. For Amber14sb, most permeation cycles can be collapsed into a single loop. Two loops, one overlapping with the loop of Amber14sb, explain most of the observed cycles in CHARMM36m simulations. Under most conditions, the rate-limiting step involves the formation of a central ion pair in S2 and S3. Water is found occasionally in S1 and S4. Our analysis suggests that, except for KcsA E71A using Amber14sb, S1 water occupancy is irrelevant to ion permeation when a positive membrane voltage is applied. Transient occupancy of a water molecule in S4 is involved in the permeation, but no water molecule passes through the SF in most cases.

Rectangular voltage pulses of 450 mV induce electroporation of a synthetic POPC bilayer in 10 μs.^62^ At extremely high voltages (450 mV or above), we observed deviations in permeation cycles from the typical ones, accompanied by a few water permeation events reminiscent of the water finger protrusion that happens before the pore formation in lipid bilayers.^48^ Simulations of KcsA E71A and TRAAK WT using CHARMM36m also reveal rare water copermeation events. There were reports of water-mediated permeation events, where ions and water molecules permeated through the channel under an applied membrane voltage. However, the effective membrane voltages in their studies were much higher than the physiologically relevant voltages,^63−66^ confirming the propensity of water permeation at high voltages. At lower voltages, direct knock-on was observed in independent studies using MD simulations.^9,10,61,67,68^

Experimental evidence appears to be in favor of direct knock-on over soft knock-on in general.^59^ For instance, single wavelength anomalous dispersion X-ray diffraction data revealed full occupancy of S1 to S4 by K^+^ in NaK2K^69^ and TREK-1,^70^ making the traditional interpretation of the electron density map as a superposition of KWKW and WKWK for S1 to S4 less appealing. Solid-state nuclear magnetic resonance measurements support the idea of water-free binding sites, at least for S3 and S4, in NaK2K.^71^ Electrophysiological measurements with Rb^+^ and Cs^+^ suggest that 3 to 4 ions are required to enter the SF to activate the filter for the voltage-dependent gating in K2P channels which lack a canonical voltage-sensing domain.^72^ That said, fitting MD snapshots to two-dimensional infrared spectroscopy data probing the occupancy of S1 to S3 in KcsA revealed that both states representing soft knock-on and states representing direct knock-on derived from MD could explain the data equally well.^9^ Despite the poor signal-to-noise ratio, different experimental conditions, and difficulties in interpreting streaming potential measurements, the data arguably support the copermeation of water through K^+^ channels and are compatible with the soft knock-on mechanism.^73−75^ More efforts are required to distinguish under what conditions one permeation mechanism overtakes the other. We believe that direct knock-on is an overall better hypothesis explaining and reconciling various independent experimental and computational findings than soft knock-on regarding ion permeation in K^+^ channels under physiological conditions.

Variations in conductance and permeation cycles were observed from MthK WT, KcsA E71A, NaK2K F92A, and TRAAK WT. It implies that the amino acid sequence of the SF is not the only determining factor for K^+^ permeation across the SF. Since ion permeation is sensitive to the precise geometry of the SF,^4^ residues in the vicinity of the SF likely contribute to the conformational dynamics of the SF and influence the movement of ions through the SF. In KcsA, E71A is a substitution of the glutamic acid behind the SF. The mutation prevents the inactivation of KcsA, possibly by disrupting the carboxyl-carboxylate interactions between D80 and E71.^44^ Such a mutation may alter the conductance of the channel by influencing the geometry of the SF, and the impacts would be reflected in the permeation cycles. The analysis framework presented in this work allows for a systematic comparison of permeation cycles in different simulated systems to study the underlying permeation mechanisms quantitatively. With our framework, studies such as exploring the permeation mechanisms used by different mutants of K^+^ channels in the future become possible.

### 4.3 Charge Strength Dependence of Permeation Cycles

It is crucial to emphasize that our findings were based on nonpolarizable fixed-charge atomistic models. Polarizable force fields, such as AMOEBA,^76^ are not as popular as the fixed-charge force fields, such as Amber14sb and CHARMM36m, in part due to higher computational cost. However, improving the existing nonpolarizable fixed-charge force fields is desirable as the simulated conductance of K^+^ channels is often underestimated.^68^ Using our simulation data, the conductances of the MthK pore at 100 mV were estimated to be 11.3 ± 0.49 pS and 7.69 ± 0.49 pS for Amber14sb and CHARMM36m, respectively. We believe our estimated conductance is approximately 1 order of magnitude lower than the experimental values.^57,58^ An alternative to introducing polarizability to fixed-charge force fields is to model the electronic polarization effects using a mean-field approximation via charge scaling, known as the electronic continuum correction (ECC).^52^ Our simulations reveal new permeation cycles with higher ion occupancy by scaling the charges of charged residues and ions to 0.7 of the original charges. For CHARMM36m, there is a 20-fold increase in the conductance of MthK WT. In line with our charge scaling simulations, it has been shown that using AMOEBA or CHARMM36m with ECC favors full ion occupation over water/ion alternate occupation in the SF, likely further promoting water-free ion permeation.^47^ While the authors computed the free energy profile for the single-vacancy mechanism,^77^ the single-vacancy permeation pathway was not necessarily the dominant pathway used by the channel, as we have shown that the permeation pathways depend on the actual implementation of the force field. The quantitative framework for analyzing permeation cycles presented in our work can serve as a complementary tool for identifying permeation pathways adopted by ion channels, which can be then used to calculate the associated free energy profiles to obtain a comprehensive view of the ion permeation processes. We expect that the conduction rates and permeation patterns will differ when switching from a fixed-charge force field to a polarizable force field.

The precise details of the ion permeation mechanism are sensitive to the choice of force field. Amber14sb and CHARMM36m, being fixed-charge and nonpolarizable force fields, result in different conduction rates and permeation pattern cycles. Despite overlapping pathways, our Markov state models reveal that the dominant, direct knock-on-based permeation pathways observed in Amber14sb and CHARMM36m simulations are not identical. Ion–protein interactions influence the ion permeation processes heavily. The standard CHARMM force field with the CHARMM water and ion models resulted in water-free direct knock-on in previous work^9,10,61,67,68,78^ and our work. However, CHARMM with a force field correction weakening Lennard-Jones interactions between ions and backbone carbonyl groups of the SF, typically termed “NBFIX” in the context of K^+^ channel simulations, led to water-mediated soft knock-on.^79−82^ Because of the parameter dependence on permeation patterns, it is highly advised, if possible, to use different force field models for comparison as far as ion permeation is concerned. The differences in permeation details between different force field models suggest that determining optimal force field parameters is of paramount importance. Our framework for analyzing permeation cycles provides additional insights into the quantitative details of ion permeation events that help guide the calibration of force field parameters for properly characterizing the ion permeation processes in MD simulations. An optimization strategy for developing a nonpolarizable force field dedicated to reproducing desired conduction properties in K^+^ channels is to match the conductance, permeation cycles, and MFPTs of permeation steps for channels simulated using a polarizable force field. From our results, applying charge scaling to CHARMM36m can be a promising starting point for this purpose.

### 4.4 Conclusions

Using molecular dynamics simulations and Markov state modeling, we computed permeation cycles representing ion permeation events in the selectivity filter of potassium channels. The permeation cycles demonstrate the robustness of the direct knock-on permeation mechanism over a wide range of conditions, including potassium concentration, temperature, and voltage. These factors primarily influence ion conduction rates, while the impacts on selective filter occupancy and permeation pathways were relatively insignificant. Water copermeation and deviations from the typical ion permeation cycles were almost only observed at supraphysiological voltages. Additional simulations suggest that direct knock-on remains the dominant permeation mechanism in different potassium channels with a highly conserved selective filter. Lastly, we show the charge strength dependence of permeation cycles via charge scaling and demonstrate the possibility of force field optimization using the framework presented in this work. Our results reveal the underlying details of permeation processes in the selectivity filter and help answer long-standing questions regarding permeation mechanisms in potassium channels.

## Supporting Information Available

Ion jumps in permeation cycles, summary of MD trajectories, algorithms for identification of permeation events and permeation cycle reduction, relaxation times of the slowest dynamics, Chapman–Kolmogorov test for relaxation in Markov chains, mean first passage times of SF occupation state transitions as a function of lag time, probability density functions of first passage times of SF occupation state transitions, net fluxes, currents observed in MD and MC simulations, Chapman–Kolmogorov test comparing transition probabilities observed from MD simulations and predicted with MSMs, potassium and water occupancy in SF binding sites, and number density for potassium and water along the *z*-axis (PDF)
