# Supplementary material for: Ion Conduction Mechanisms in Potassium Channels Revealed by Permeation Cycles
Source: J Chem Theory Comput. 2023 Apr 11;19(9):2574–89. doi: 10.1021/acs.jctc.3c00061 (PMC10173462; doi:10.1021/acs.jctc.3c00061)
Supplement: Supplementary file 1 — ct3c00061_si_001.pdf [file ct3c00061_si_001.pdf]

Supporting Information: Ion Conduction Mechanisms in Potassium Channels Revealed by Permeation Cycles

Chun Kei Lam and Bert L. de Groot*

Computational Biomolecular Dynamics Group, Max Planck Institute for Multidisciplinary Sciences, Göttingen, Germany

E-mail: bgroot@gwdg.de

Supporting Information Available

1 Ion Jumps in Permeation Cycles

In addition to the instantaneous SF occupation states, we kept track of the number of net ion jumps $j_k(t)$ at time t during the SF occupation state transitions. In our convention, only ion jumps to or from S1, S2, S3, or S4 are considered (Figure S1A). For example, there is a positive ion jump ($j_k = +1$) when an ion hops to the next binding site toward S0. Multiple ions hopping at the same time is possible. Water jumps $j_w(t)$ are also counted using the same convention. A complete permeation cycle is achieved by returning to the initial occupation state after time T with $\sum_{t^*=t}^{t+T-1} j_k(t^*) = +5$ net K^+ jumps in total.

Two possible transitions for C0KK0C → CK0KKW are illustrated in Figure S1B. The first transition involves two forward ion jumps, one from S2 to S1 and one from Scav to S4. This transition is relevant to the permeation cycles at a positive membrane potential.

Table S1: Summary of MD trajectories simulated using Amber14sb.

name	K^+ (M)	T (K)	V (V)	$\frac{q}{q_0}$	# sims	t (ns)	# K^+ event	# water event
MthK WT	0.1	323	0.3	1.0	10	500	39,32,41,44,29, 32,14,34,15,48	0,0,0,0,0, 0,0,0,0,0
MthK WT	0.5	323	0.3	1.0	10	500	39,50,63,45,51, 30,32,66,54,46	0,0,0,0,0, 0,0,0,0,0
MthK WT	1.0	323	0.3	1.0	20	500	52,45,59,41,26, 27,52,63,72,22, 42,29,68,34,64, 57,52,51,50,58	0,0,0,0,0, 0,0,0,0,0, 0,0,0,0,0, 0,0,0,0,0
MthK WT	2.0	323	0.3	1.0	10	500	53,8,75,35,63, 29,59,58,26,36	0,0,0,0,0, 0,0,0,0,0
MthK WT	1.0	283	0.3	1.0	10	500	6,3,9,16,4, 3,14,2,2,6	0,0,0,0,0, 0,0,0,0,0
MthK WT	1.0	293	0.3	1.0	10	500	3,19,1,14,7, 1,13,13,13,13	0,0,0,0,0, 0,0,0,0,0
MthK WT	1.0	303	0.3	1.0	10	500	3,29,18,28,14, 18,27,6,25,36	0,0,0,0,0, 0,0,0,0,0
MthK WT	1.0	313	0.3	1.0	10	500	55,38,37,30,22, 16,32,37,27,13	0,0,0,0,0, 0,0,0,0,0
MthK WT	1.0	333	0.3	1.0	10	500	53,49,80,78,63, 73,75,59,56,87	0,0,0,0,0, 0,0,0,0,0
MthK WT	1.0	323	0.05	1.0	10	500	5,1,3,1,1,2,2,0,0,0, 0,0,1,1,0,3,2,5,0,1	0,0,0,0,0, 0,0,0,0,0, 0,0,0,0,0, 0,0,0,0,0
MthK WT	1.0	323	0.1	1.0	10	500	1,3,5,3,3,4,2,7,3,2, 2,0,3,0,1,6,3,2,4,5, 3,5,9,4,1,6,6,5,7,1	0,0,0,0,0, 0,0,0,0,0, 0,0,0,0,0, 0,0,0,0,0, 0,0,0,0,0, 0,0,0,0,0
MthK WT	1.0	323	0.2	1.0	10	500	19,14,15,34,20, 6,21,14,18,24	0,0,0,0,0, 0,0,0,0,0
MthK WT	1.0	323	0.45	1.0	10	500	82,92,106,112,97, 118,96,83,82,140	0,0,0,0,1, 0,0,0,0,0
MthK WT	1.0	323	0.6	1.0	10	500	115,109,77,88,97, 97,132,108,127,100	2,2,0,0,0, 0,1,1,0,0

Table S2: Summary of MD trajectories simulated using Amber14sb (continue).

name	K^+ (M)	T (K)	V (V)	$\frac{q}{q_0}$	# sims	t (ns)	# K^+ event	# water event
KcsA E71A	1.0	323	0.3	1.0	20	500	19,33,37,56,30, 28,36,35,23,30, 37,34,39,24,39, 30,48,32,32,34	0,0,0,0,0, 0,0,0,0,0, 0,0,0,0,0, 0,0,0,0,0
NaK2K F92A	1.0	323	0.3	1.0	20	500	9,17,3,20,22, 27,30,20,26,31, 18,23,8,19,22, 27,23,19,22,19	0,0,0,0,0, 0,0,0,0,0, 0,0,0,0,0, 0,0,0,0,0
TRAAK WT	1.0	323	0.3	1.0	20	500	6,7,20,2,20, 7,13,8,2,7, 9,14,6,9,8, 9,7,11,7,9	0,0,0,0,0, 0,0,0,0,0, 0,0,0,0,0, 0,0,0,0,0
MthK WT	1.0	323	0.3	0.7	10	500	3,1,2,7,3,4,4,4,8,1	0,0,0,0,0, 0,0,0,0,0
MthK WT	1.0	323	0.3	0.75	10	500	28,22,31,22,22, 22,33,13,21,25	0,0,0,0,0, 0,0,0,0,0
MthK WT	1.0	323	0.3	0.8	10	500	50,31,46,44,55, 41,54,58,49,46	0,0,0,0,0, 0,0,0,0,0
MthK WT	1.0	323	0.3	0.85	10	500	33,67,13,56,64, 46,44,64,20,70	0,0,0,0,0, 0,0,0,0,0
MthK WT	1.0	323	0.3	0.9	10	500	25,24,36,13,11, 45,25,9,17,3	0,0,0,0,0, 0,0,0,0,0
MthK WT	1.0	323	0.3	0.95	10	500	13,14,20,7,11, 2,10,12,6,3	0,0,0,0,0, 0,0,0,0,0

Table S3: Summary of MD trajectories simulated using CHARMM36m.

name	K^+ (M)	T (K)	V (V)	$\frac{q}{q_0}$	# sims	t (ns)	# K^+ event	# water event
MthK WT	0.1	323	0.3	1.0	10	500	19,29,4,19,11, 6,9,10,8,23	0,0,0,0,0, 0,0,0,0,0
MthK WT	0.5	323	0.3	1.0	10	500	3,12,0,16,37, 14,8,22,12,23	0,0,0,0,0, 0,0,0,0,0
MthK WT	1.0	323	0.3	1.0	20	500	48,40,17,7,24, 25,19,14,36,3, 16,11,32,9,16, 17,49,8,24,10	0,0,0,0,0, 0,0,0,0,0, 0,0,0,0,0, 0,0,0,0,0
MthK WT	2.0	323	0.3	1.0	10	500	28,45,35,21,23, 20,21,28,18,22	1,0,0,0,0, 0,0,0,0,0
MthK WT	1.0	283	0.3	1.0	10	500	6,3,3,10,10, 11,11,1,3,16	0,0,0,0,0, 0,0,0,0,0
MthK WT	1.0	293	0.3	1.0	10	500	12,17,9,0,11, 2,27,6,13,9	0,0,0,0,0, 0,0,0,0,0
MthK WT	1.0	303	0.3	1.0	10	500	19,18,12,2,3, 15,13,4,20,43	0,0,0,0,0, 0,0,0,0,0
MthK WT	1.0	313	0.3	1.0	10	500	22,16,19,40,9, 2,22,23,5,4	0,0,0,0,0, 0,0,0,0,0
MthK WT	1.0	333	0.3	1.0	10	500	1,55,36,53,12, 27,14,41,39,7	0,0,0,0,0, 0,0,0,0,0
MthK WT	1.0	323	0.05	1.0	10	500	0,0,0,0,0,0,0,0,2,0, 0,0,0,0,0,0,1,0,0,0	0,0,0,0,0, 0,0,0,0,0, 0,0,0,0,0, 0,0,0,0,0
MthK WT	1.0	323	0.1	1.0	10	500	6,0,2,5,6,5,1,1,5,2, 1,2,1,8,3,5,1,0,0,0, 1,1,1,1,1,2,0,3,5,3	0,0,0,0,0, 0,0,0,0,0, 0,0,0,0,0, 0,0,0,0,0, 0,0,0,0,0, 0,0,0,0,0
MthK WT	1.0	323	0.2	1.0	10	500	7,6,2,5,0,6,6,4,16,9	0,0,0,0,0, 0,0,0,0,0
MthK WT	1.0	323	0.45	1.0	10	500	44,5,32,42,32, 29,28,15,28,29	0,0,0,0,1, 1,0,0,0,1
MthK WT	1.0	323	0.6	1.0	10	500	12,26,23,30,23, 31,28,7,14,14	1,1,0,0,0, 1,2,1,0,0

Table S4: Summary of MD trajectories simulated using CHARMM36m (continue).

name	K^+ (M)	T (K)	V (V)	$\frac{q}{q_0}$	# sims	t (ns)	# K^+ event	# water event
KcsA E71A	1.0	323	0.3	1.0	20	500	26,28,20,37,8,<br>35,12,26,28,23,<br>25,41,31,41,18,<br>25,26,23,14,28	0,0,0,0,0,<br>0,0,0,0,0,<br>0,0,2,0,0,<br>0,0,2,0,0
NaK2K F92A	1.0	323	0.3	1.0	20	500	30,32,36,40,32,<br>35,17,37,38,30,<br>24,31,39,33,38,<br>22,31,33,27,27	0,0,0,0,0,<br>0,0,0,0,0,<br>0,0,0,0,0,<br>0,0,0,0,0
TRAAK WT	1.0	323	0.3	1.0	20	500	15,11,12,13,0,<br>10,26,16,9,15,<br>23,12,16,14,2,<br>6,14,4,1,30	1,0,0,0,0,<br>0,1,0,0,0,<br>0,0,0,0,0,<br>0,0,0,0,0
MthK WT	1.0	323	0.3	0.7	10	500	322,391,463,460,465,<br>520,426,294,447,545	0,0,0,0,0,<br>0,0,0,0,0
MthK WT	1.0	323	0.3	0.75	10	500	192,204,163,91,206,<br>127,149,136,278,222	0,0,0,0,0,<br>0,0,0,0,0
MthK WT	1.0	323	0.3	0.8	10	500	25,18,31,17,12,<br>10,15,13,50,49	0,0,0,0,0,<br>0,0,0,0,0
MthK WT	1.0	323	0.3	0.85	10	500	7,4,2,12,0,2,2,2,2,4	0,0,0,0,0,<br>0,0,0,0,0
MthK WT	1.0	323	0.3	0.9	10	500	2,0,3,2,0,1,1,0,1,0	0,0,0,0,0,<br>0,0,0,0,0
MthK WT	1.0	323	0.3	0.95	10	500	0,1,1,1,1,3,4,3,0,8	0,0,0,0,0,<br>0,0,0,0,0

Data: SF occupancy $s[t]$ at time t in a MD trajectory of length L, number of net ion jumps $j_k[t]$ at time t of length $L-1$, chosen initial and final state S_c of the cycles

Result: list l of sub-trajectories $e_i[t']$, each representing an independent permeation cycle of length T_i

$l \leftarrow$ empty list;
$t \leftarrow 0$;
while $t < L-1$ **do**
  if $s[t] = S_c$ **then**
    $T \leftarrow 0$;
    $found \leftarrow$ **false**;
    while $T < L-t-1$ ***and*** $found =$ ***false*** **do**
      $T \leftarrow T+1$;
      if $s[t+T] = S_c$ **then**
        `/* compute cumulative net K+ jumps between t and t+T */`
        $n_k \leftarrow \sum_{t'=t}^{t'=t+T-1} j_k[t']$;
        if $n_k = 5$ **then**
          `/* five net ion jumps and returning to the initial state make one cycle */`
          append $s[t:t+T+1]$ to l;
          $found \leftarrow$ **True**;
        else if $n_k \leq -5$ ***or*** $n_k \geq 10$ **then**
          `/* not save it as the sub-trajectory contains more than one cycle */`
          $found \leftarrow$ **True**;
    end
    $t \leftarrow t+T-1$;
  end
  $t \leftarrow t+1$;
end

Algorithm 1: Identification of permeation events. See Figure S1 for the explanation of the number of net ion jumps j_k.

```
Data: sub-trajectory e_i[t] expressing one independent permeation cycle of length T_i,
      and number of net ion jumps j_{k,i}[t] at time t of length T_i − 1 corresponding
      to e_i[t]
Result: simplified sub-trajectory e_r containing one reduced permeation cycle
t ← 0;
e_r ← empty list;
while t < T_i − 1 do
    s_t ← e_i[t];
    if s_t not in e_r then
        append s_t to e_r;
        I ← { t' | e_i[t'] = s_t };
        sort I in descending order;
        foreach t' ∈ I do
            if Σ_{t''=t}^{t'-1} j_{k,i}[t''] = 0 then
                /* skip transitions that eventually return to the initial
                   state with no net ion jump                              */
                t ← t';
                break;
            end
        end
    end
    t ← t + 1;
end
append e_i[T_i] to e_r;
```

Algorithm 2: Permeation cycle reduction. See Figure S1 for the explanation of the number of net ion jumps j_k.

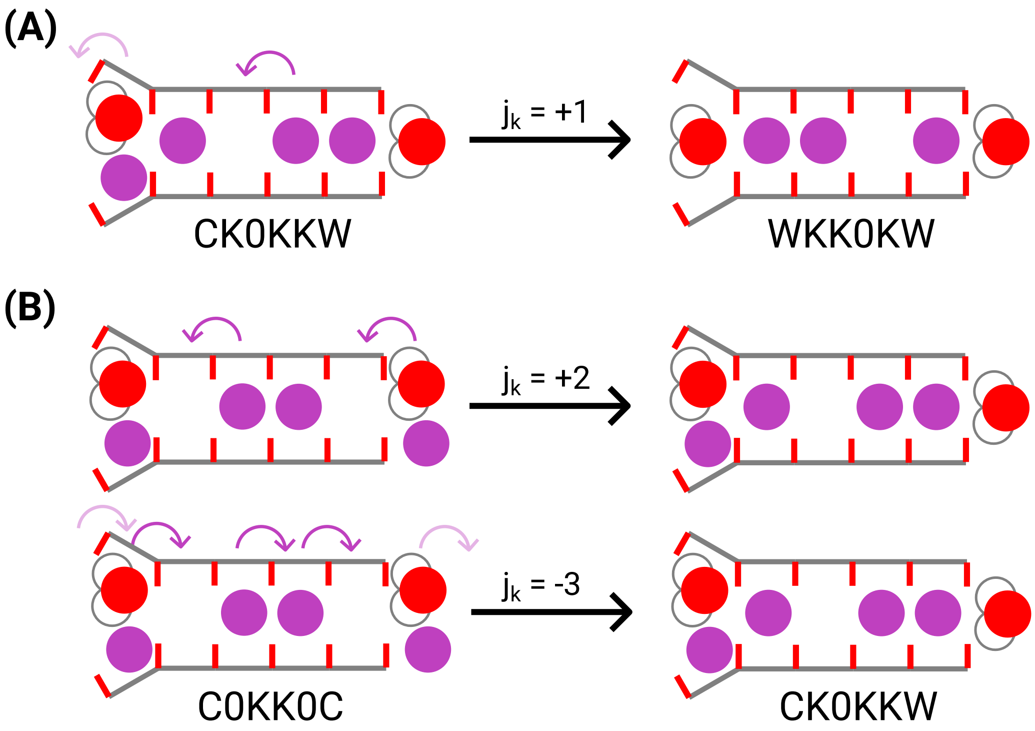

Figure S1: (A) Definition of ion jumps j_k. (B) Example illustrating necessity of specifying ion jumps j_k.

However, the second one, where three backward ion jumps are involved (the jumps from Scav to the cavity of the channel and from the extracellular side to S0 are excluded), is also possible. It implies that using only SF occupation states cannot describe permeation events uniquely. Therefore, we specified ion jumps $j_k(t)$ and water jumps $j_w(t)$ in the MSMs to avoid ambiguity.

2 Validation of Markov State Models

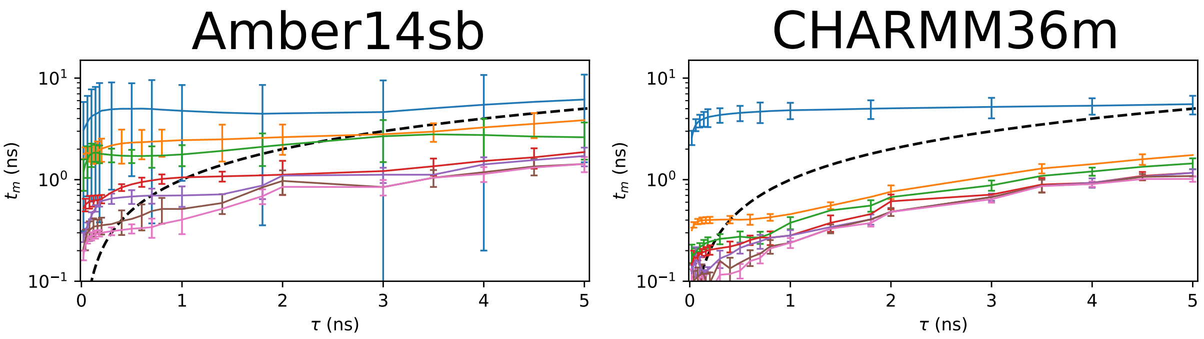

Figure S2: Relaxation time t_m of slowest dynamics ($2 \leq m \leq 7$) as function of lag time τ for MthK WT simulated at 323 K and 300 mV in 1 M KCl solution using Amber14sb and CHARMM36m. Errors are the normal-based 95% bootstrap intervals ($B = 100$). In each bootstrap iteration, MD trajectories were resampled with replacement to compute a transition matrix for estimating eigenvalues via eigendecomposition.

For an ideal MSM, the relaxation time t_m is a constant, independent of the choice of lag time τ. The relaxation times t_m for $m > 2$ increases with τ because τ is comparable to or longer than the relaxation times, therefore overestimating the timescales (Figure S2). We argue that using the convergence of the first M relaxation times to evaluate the deviation from the ideal Markov process is not comprehensive. It rests on the assumption that the motions of interest are the slowest motions in the simulated system. This assumption, however, does not hold in the context of ion permeation, as a complete ion permeation event consists of processes occurring on a wide range of timescales from tens of picoseconds to tens of nanoseconds. The fastest permeation step WK0KKW $\rightarrow$ WKK0KW has a MFPT of 30 ps and 50 ps for Amber14sb and CHARMM36m, respectively. Using a large τ results in an overestimation of the timescales of fast processes. Furthermore, inspecting components in the eigenvectors reveals that the slowest motion ($m = 2$) in AMBER simulations is irrelevant to ion permeation as this motion refers to the transitions between typical conductive states, such as WKK0KW, C0KK0C, and WK0KKW, and non-conductive states with S1 filled with water, such as CWK0KW and CWK0KC, that are absent in the permeation cycles. As a result, we did not choose the value of τ only based on the convergence of the first few

relaxation times.

We instead performed the Chapman-Kolmogorov test via observing the relaxation in the Markov chain (MC) simulations based on the transition matrices derived from the MD data (Figures S3 and S4). We evaluated the probability of staying in one of the most frequently observed SF occupation states, given that the system started in the same state. This is a harsh test for Markovianity, as a thorough sampling of transitions into and out of the metastable states is required.[S1,S2] Furthermore, we compared the transition probabilities observed from MD and predicted by the MSMs using $\tau = 20$ ps (Figures S14 and S15). Since a lag time as small as 20 ps can reproduce the MD results reasonably well, τ was chosen to be 20 ps for all MSMs. A small τ is preferable to reduce the overestimation of the mean first passage times of fast permeation steps (Figure S5), and $\tau = 20$ ps reproduces the ionic currents very well (Figure S7 and S8).

References

(S1) Prinz, J.-H.; Wu, H.; Sarich, M.; Keller, B.; Senne, M.; Held, M.; Chodera, J. D.; Schütte, C.; Noé, F. Markov models of molecular kinetics: Generation and validation. *J. Chem. Phys.* **2011**, *134*, 174105.

(S2) Paul, F.; Wu, H.; Vossel, M.; de Groot, B. L.; Noé, F. Identification of kinetic order parameters for non-equilibrium dynamics. *J. Chem. Phys.* **2019**, *150*, 164120.

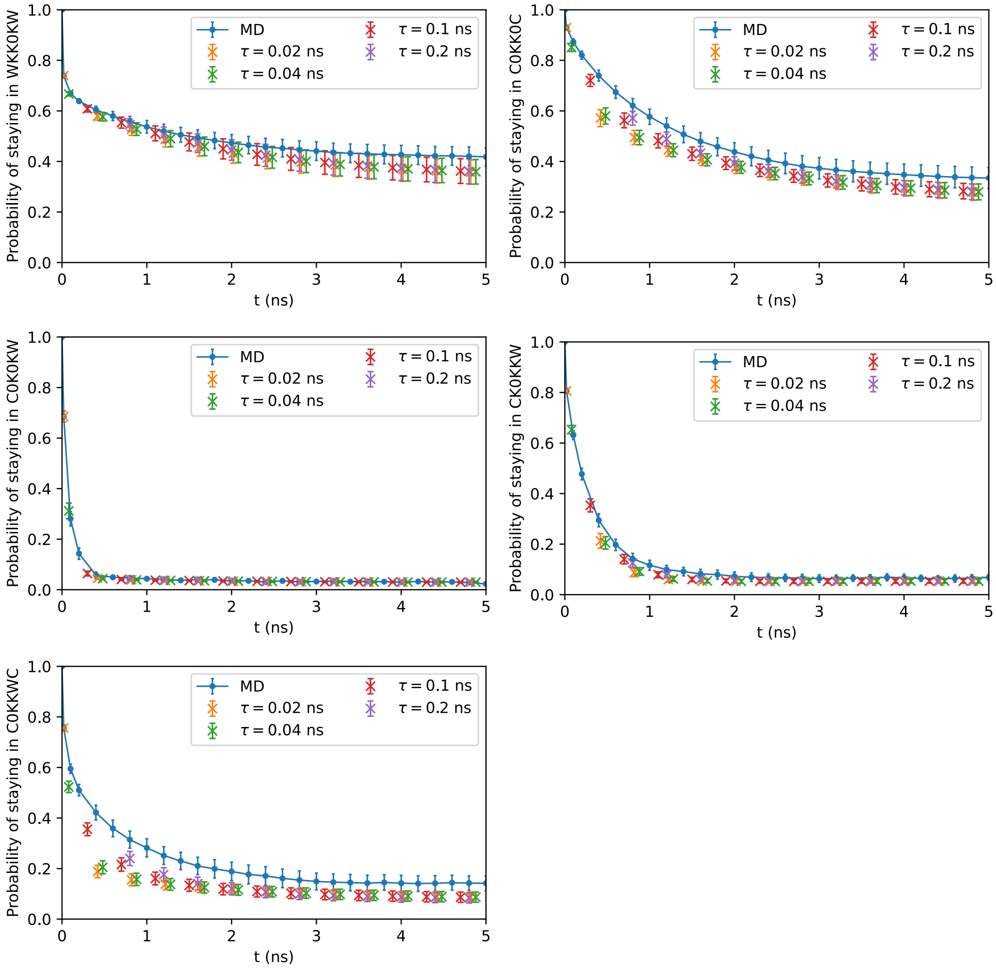

Figure S3: Chapman-Kolmogorov test for relaxation in Markov chains for MthK WT simulated at 300mV and 323 K in 1 M KCl using Amber14sb. The five states, in which the first three most frequently observed states in MD are also included, involved in most of the observed permeation cycles were chosen. Errors represent 95% normal-based bootstrap intervals ($B = 100$). In each bootstrap iteration, MD trajectories were resampled with replacement to compute a transition matrix used by the MC simulation for the estimation of the probabilities.

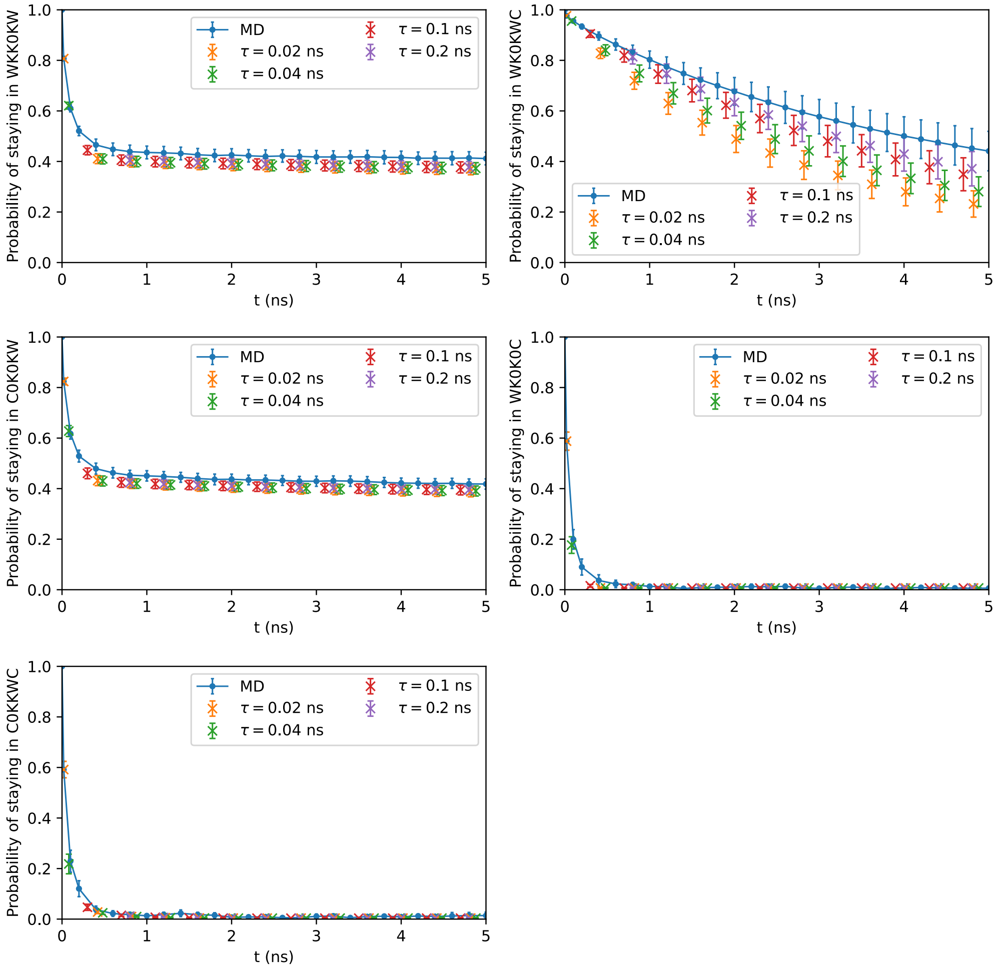

Figure S4: Chapman-Kolmogorov test for relaxation in Markov chains for MthK WT simulated at 300mV and 323 K in 1 M KCl using CHARMM36m. The five states, in which the first three most frequently observed states in MD are also included, involved in most of the observed permeation cycles were chosen. Errors represent 95% normal-based bootstrap intervals ($B = 100$). In each bootstrap iteration, MD trajectories were resampled with replacement to compute a transition matrix used by the MC simulation for the estimation of the probabilities.

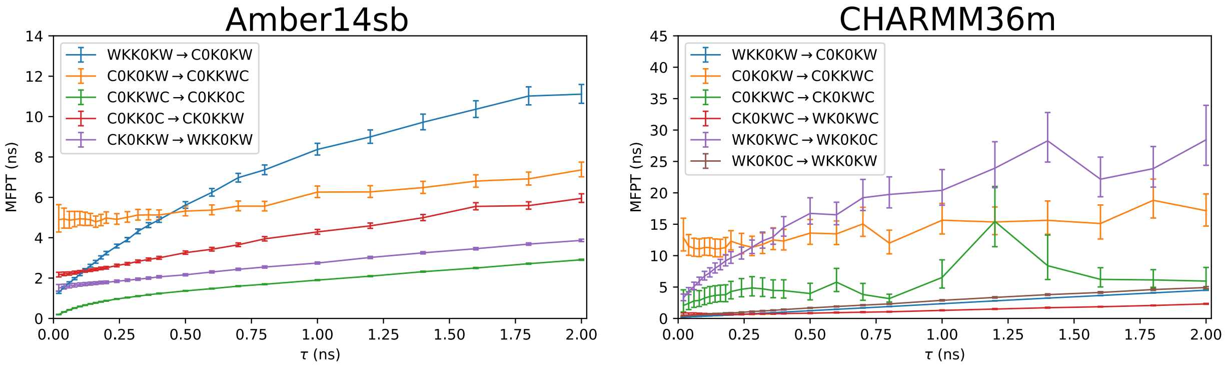

Figure S5: Mean first passage times of SF occupation state transitions as function of lag time τ for MthK WT simulated at 300mV and 323 K in 1 M KCl. Errors of MFPTs are bias-corrected and accelerated (BCa) 95% bootstrap intervals ($B = 10000$) computed from the observed values of first passage times in MD simulations.

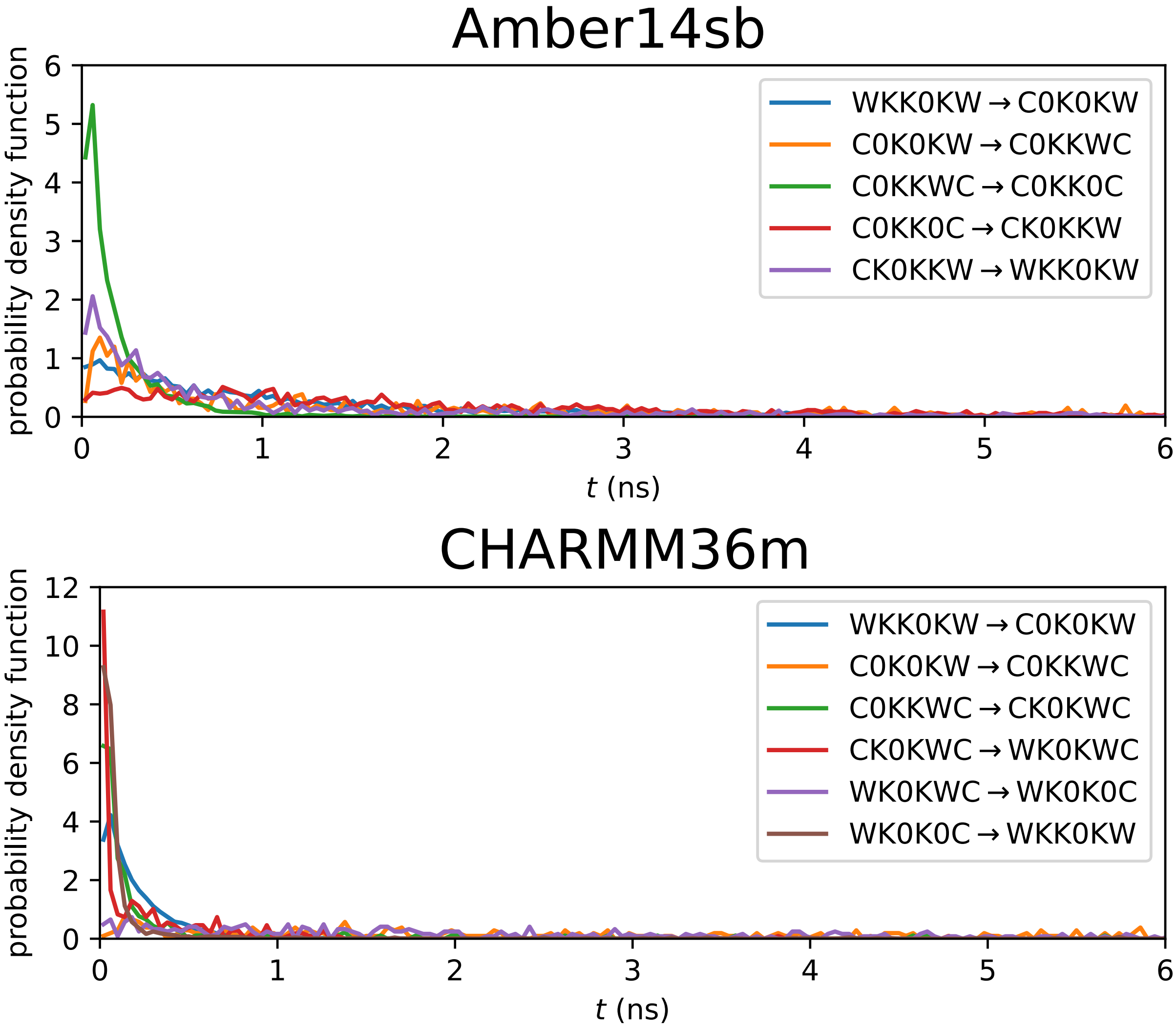

Figure S6: Probability density functions of first passage times of SF occupation state transitions for MthK WT simulated at 300mV and 323 K in 1 M KCl.

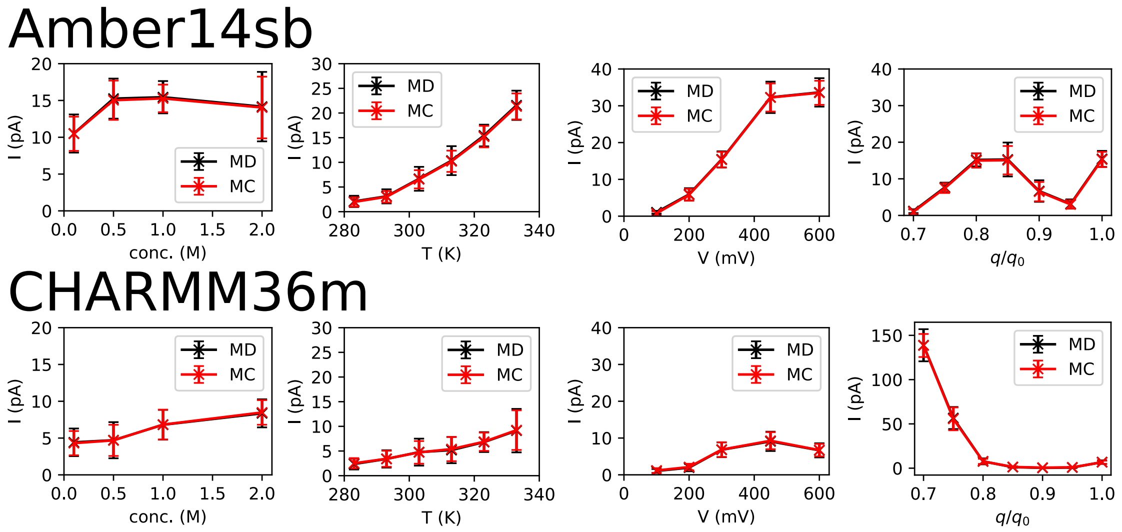

Figure S7: Currents observed in MD and MC simulations for MthK WT simulated at 300mV and 323 K in 1 M KCl. Uncertainty for the MD simulations is the 95% confidence intervals using the t-distribution. Uncertainty for the MC simulations is the 95% normal-based bootstrap intervals ($B = 100$). In each bootstrap iteration, MD trajectories were resampled with replacement to compute a transition matrix used by the MC simulation for the estimation of the currents.

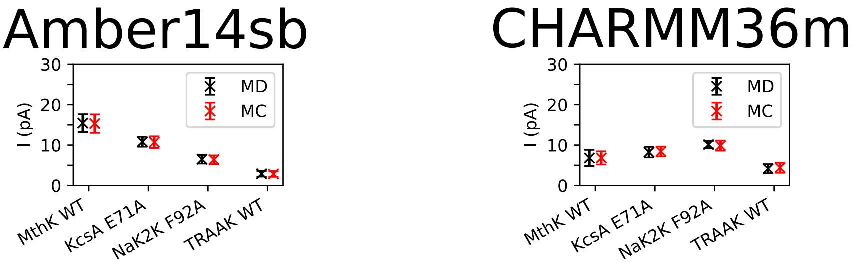

Figure S8: Currents in MD and MC simulations for MthK WT, KcsA E71A, NaK2K F92A, and TRAAK WT simulated at 300mV and 323 K in 1 M KCl. Uncertainty for the MD simulations is the 95% confidence intervals using the t-distribution. Uncertainty for the MC simulations is the 95% normal-based bootstrap intervals ($B = 100$). In each bootstrap iteration, MD trajectories were resampled with replacement to compute a transition matrix used by the MC simulation for the estimation of the currents.

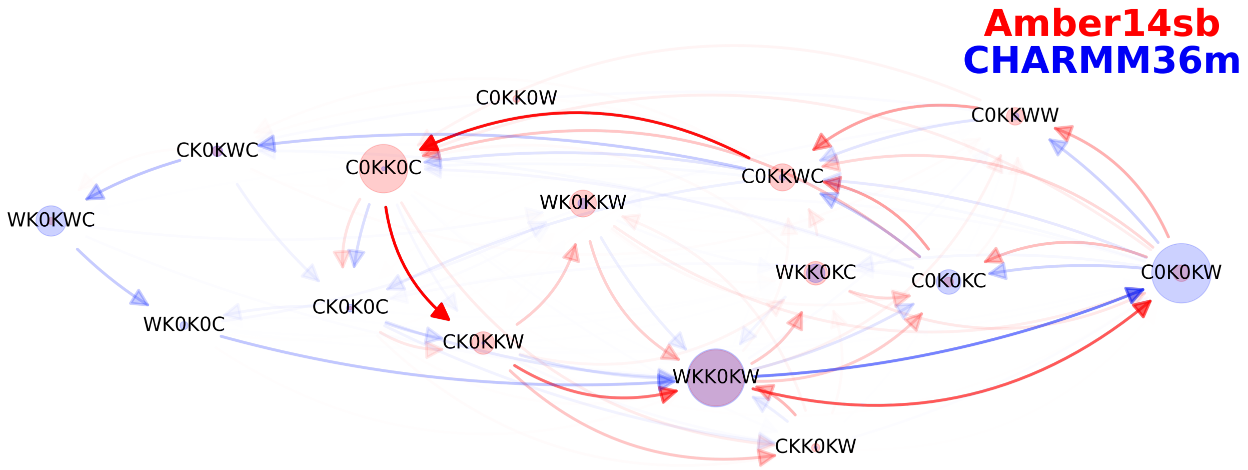

Figure S9: Net fluxes between SF occupation states for MthK WT at 300 mV and 323 K in 1 M KCl. Node sizes scale with the steady-state distributions of the SF occupation states. Edges represent the net fluxes between states. Only the net fluxes larger than 0.1 of the maximum among all net fluxes are shown.

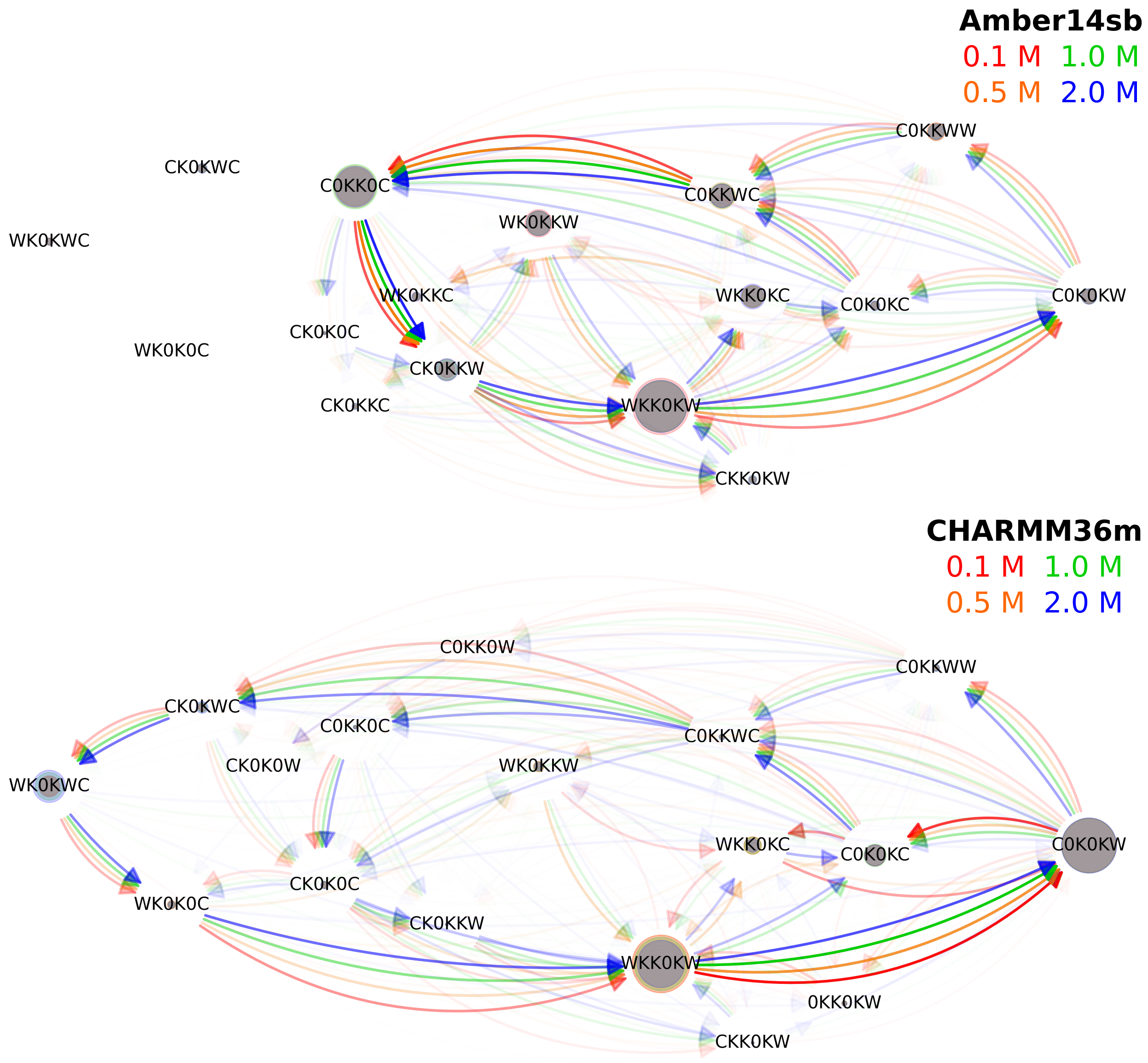

Figure S10: Net fluxes between SF occupation states for MthK WT at 300 mV and 323 K in KCl of different concentrations. Node sizes scale with the steady-state distributions of the SF occupation states. Edges represent the net fluxes between states. Only the net fluxes larger than 0.1 of the maximum among all net fluxes are shown.

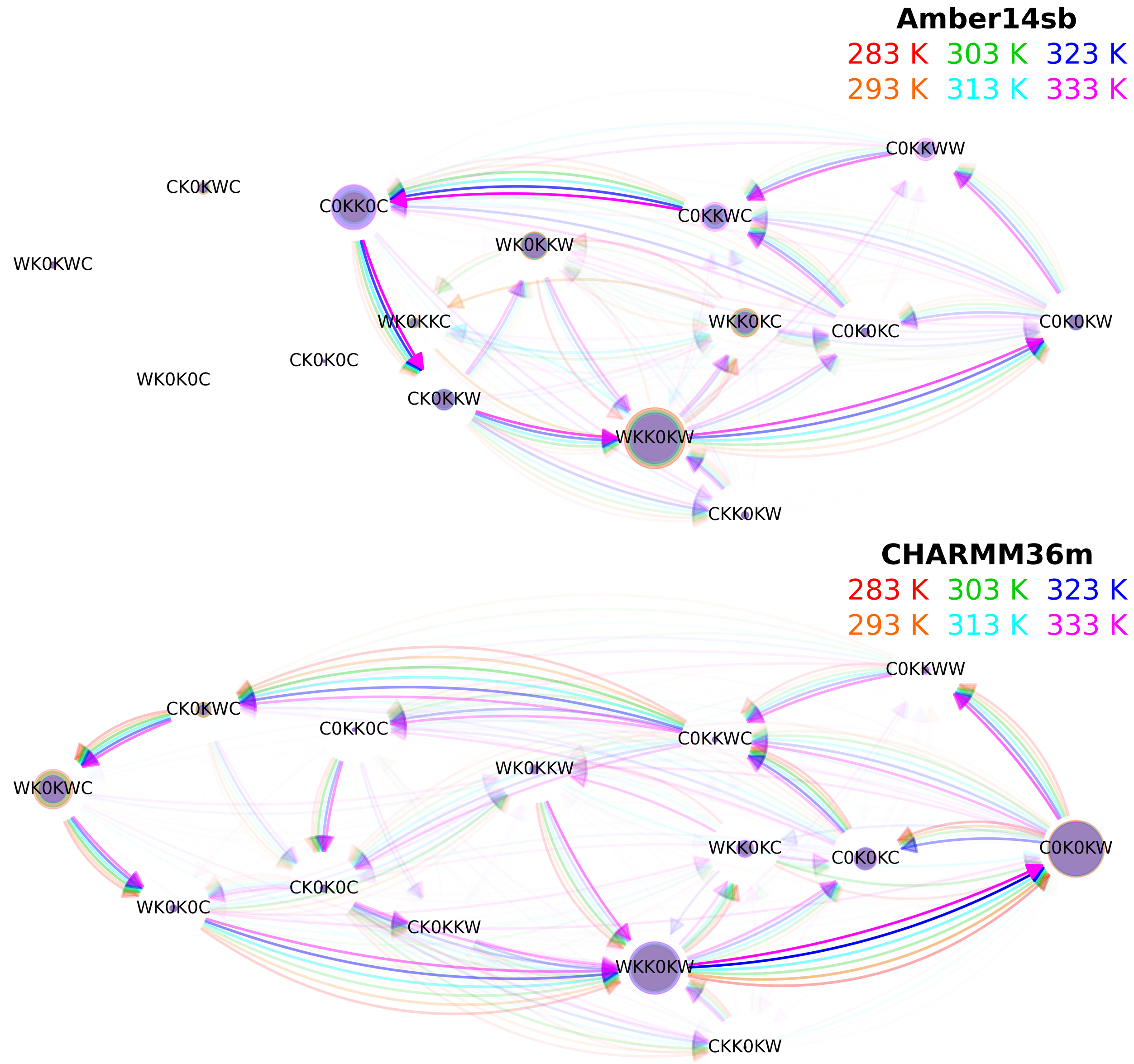

Figure S11: Net fluxes between SF occupation states for MthK WT at 300 mV and different temperature in 1 M KCl. Node sizes scale with the steady-state distributions of the SF occupation states. Edges represent the net fluxes between states. Only the net fluxes larger than 0.1 of the maximum among all net fluxes are shown.

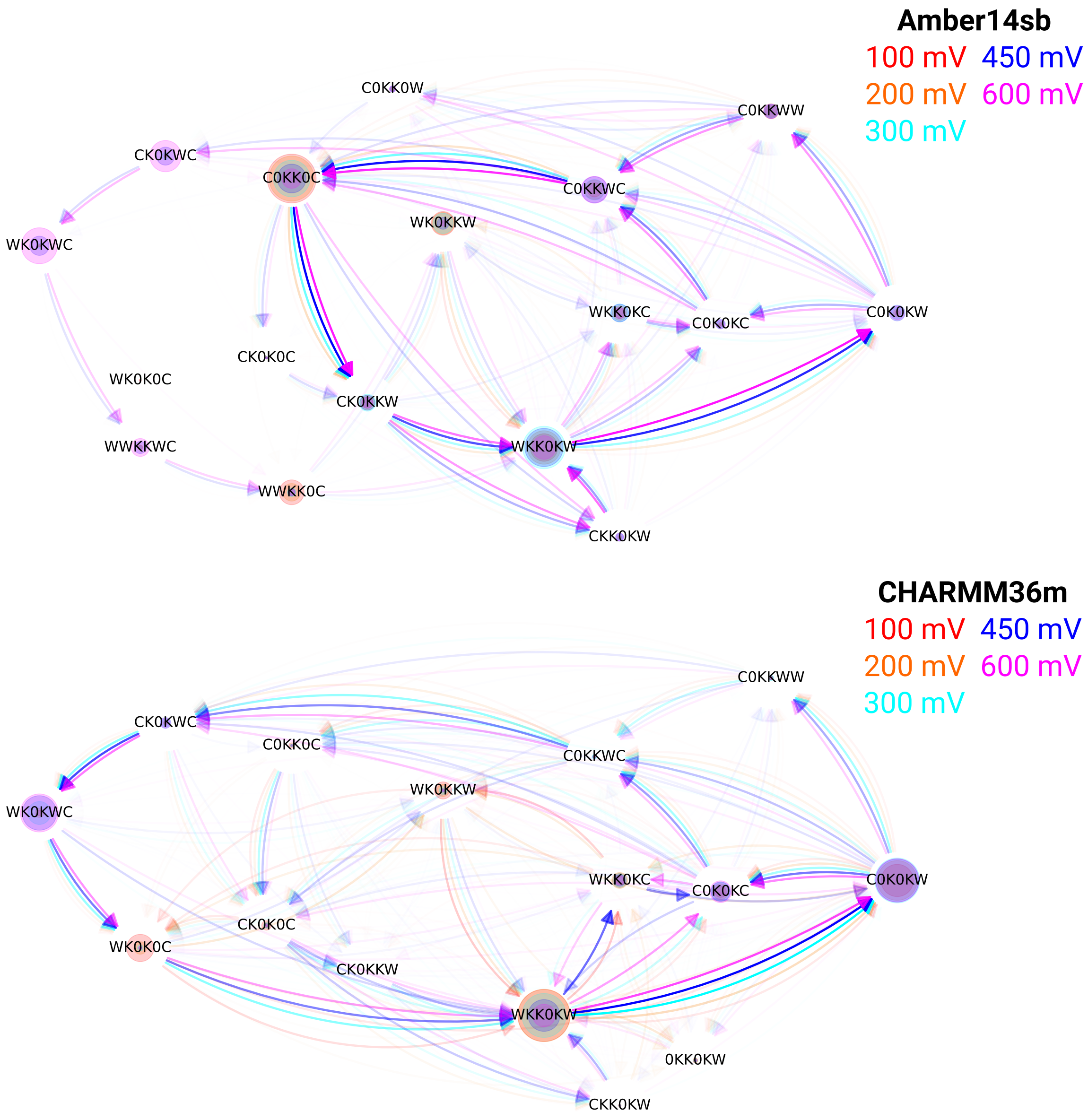

Figure S12: Net fluxes between SF occupation states for MthK WT at different membrane voltage and 323 K in 1 M KCl. Node sizes scale with the steady-state distributions of the SF occupation states. Edges represent the net fluxes between states. Only the net fluxes larger than 0.1 of the maximum among all net fluxes are shown.

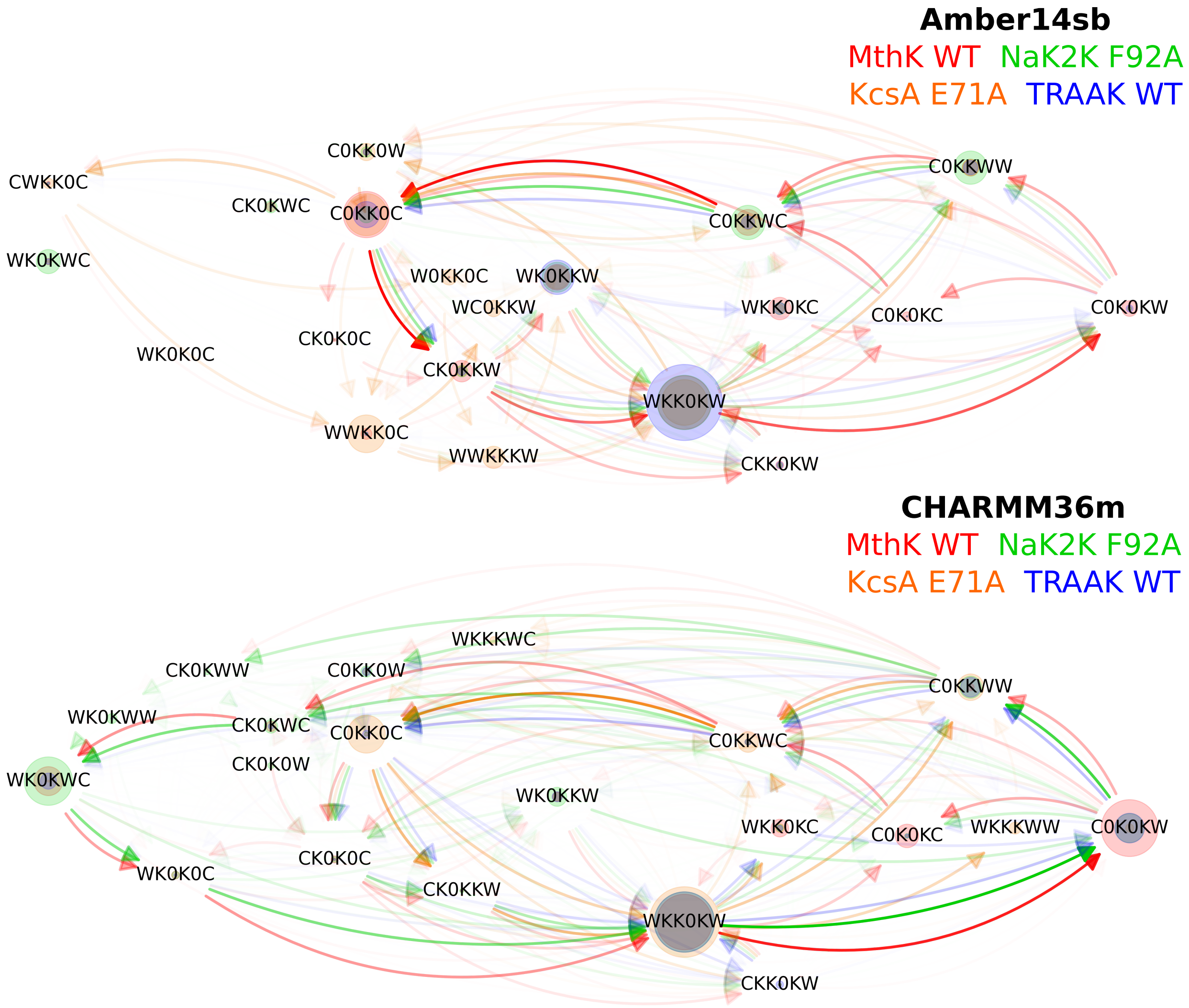

Figure S13: Net fluxes between SF occupation states for MthK WT, KcsA E71A, NaK2K F92A, and TRAAK WT at 300 mV and 323 K in 1 M KCl. Node sizes scale with the steady-state distributions of the SF occupation states. Edges represent the net fluxes between states. Only the net fluxes larger than 0.1 of the maximum among all net fluxes are shown.

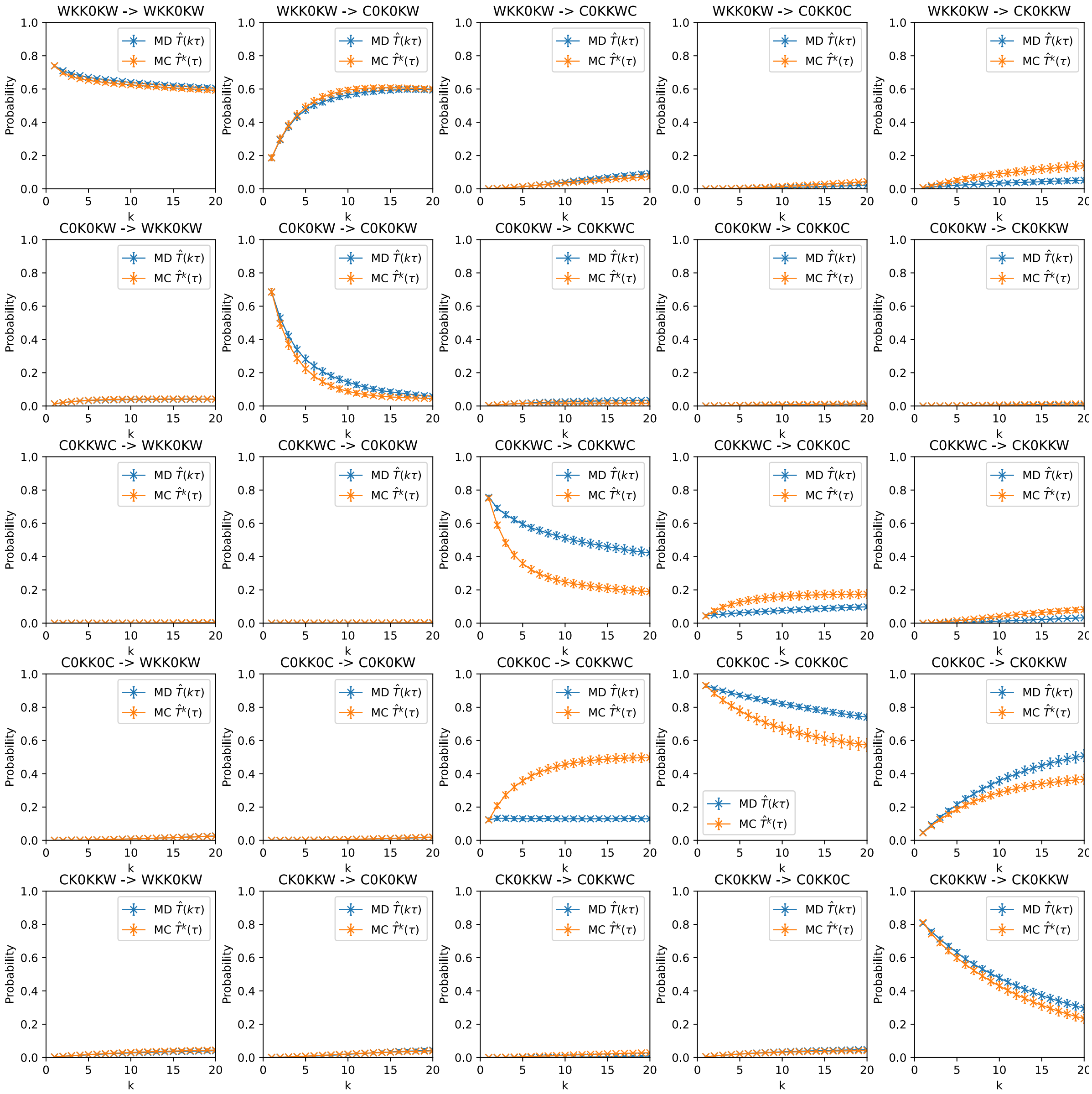

Figure S14: Chapman-Kolmogorov test comparing transition probabilities observed from MD simulations and predicted with MSMs with $\tau = 20$ ps for MthK WT simulated at 300mV and 323 K in 1 M KCl using Amber14sb. The five states, in which the first three most frequently observed states in MD are also included, involved in most of the observed permeation cycles were chosen. Errors represent 95% normal-based bootstrap intervals ($B = 100$). In each bootstrap iteration for the uncertainty of MC simulations, MD trajectories were resampled with replacement to compute the transition matrix $\hat{\boldsymbol{T}}^k(\tau)$, for $k = 1, 2, ..., 20$.

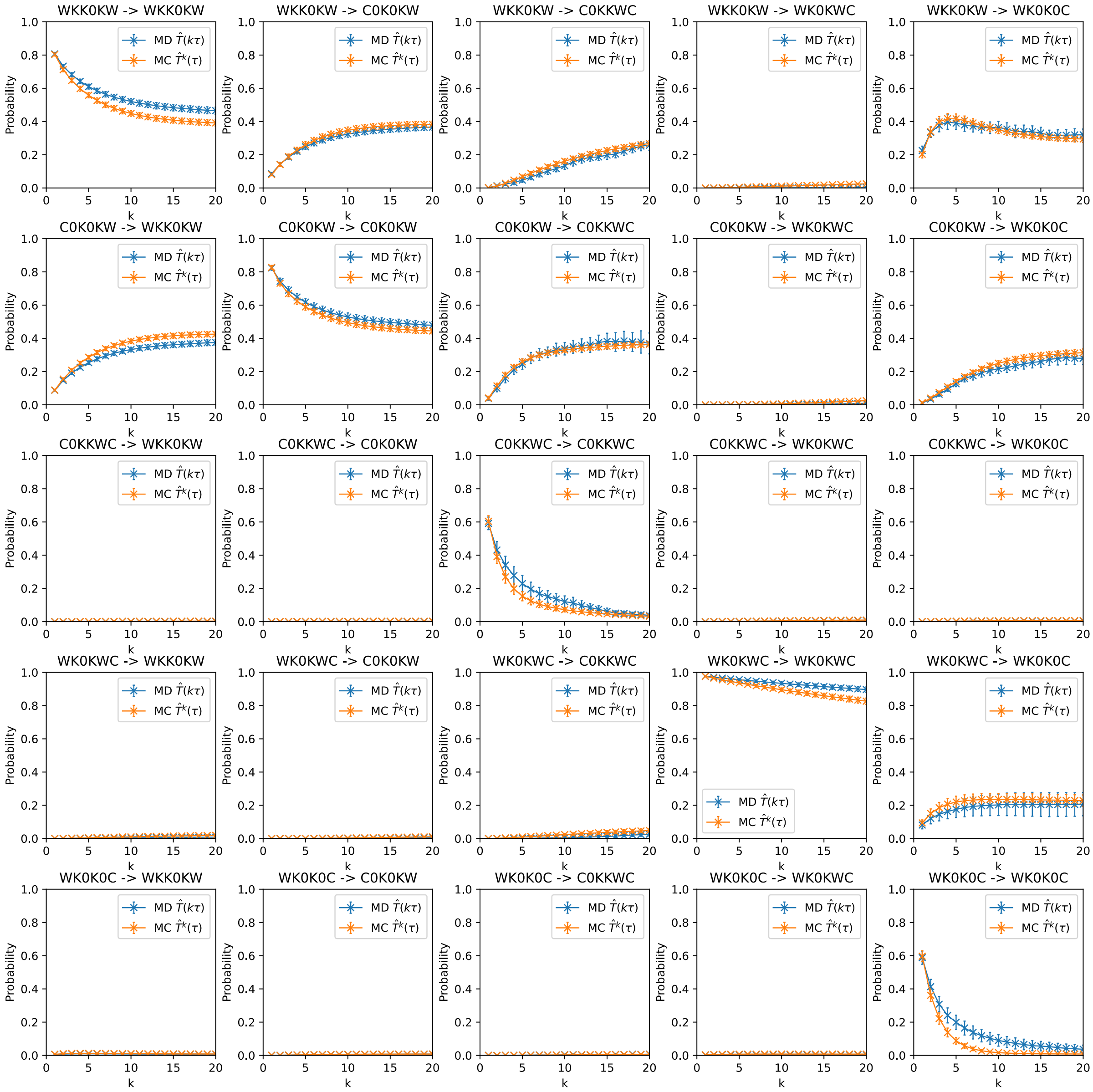

Figure S15: Chapman-Kolmogorov test comparing transition probabilities observed from MD simulations and predicted with MSMs with $\tau = 20$ ps for MthK WT simulated at 300mV and 323 K in 1 M KCl using CHARMM36m. The five states, in which the first three most frequently observed states in MD are also included, involved in most of the observed permeation cycles were chosen. Errors represent 95% normal-based bootstrap intervals ($B = 100$). In each bootstrap iteration for the uncertainty of MC simulations, MD trajectories were resampled with replacement to compute the transition matrix $\hat{\boldsymbol{T}}^k(\tau)$, for $k = 1, 2, ..., 20$.

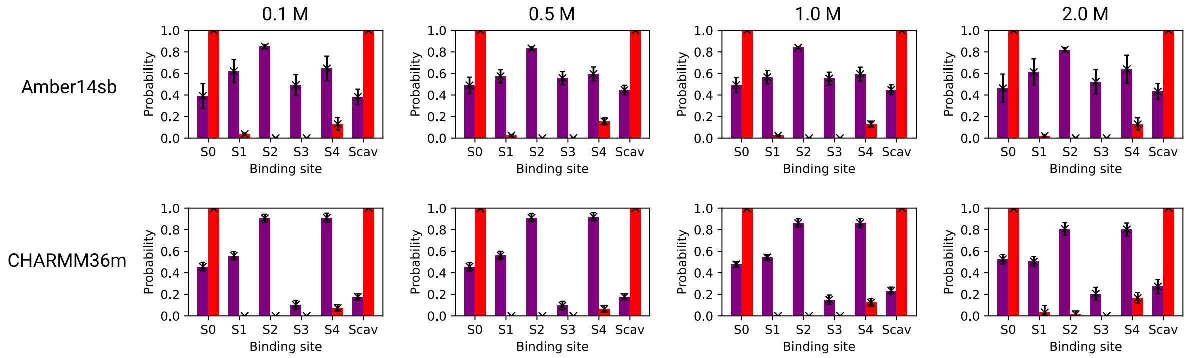

Figure S16: K^+ (purple) and water (red) occupancy in SF binding sites of MthK WT simulated at 323 K and 300 mV in KCl of different concentrations. Errors are 95% confidence intervals based on the t-distribution, with the number of observations equal to the number of independent trajectories.

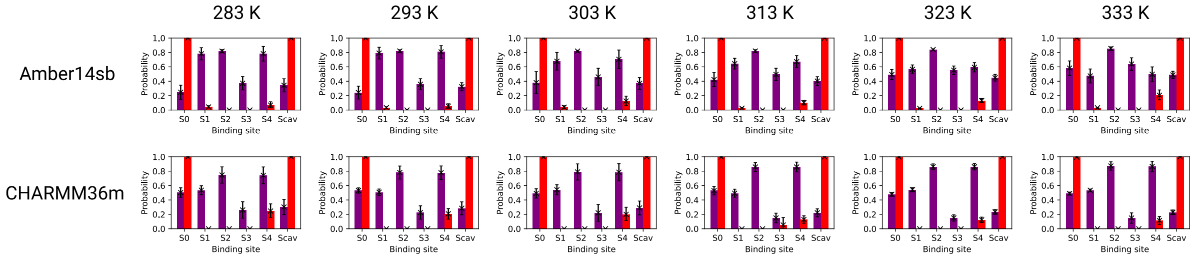

Figure S17: K^+ (purple) and water (red) occupancy in SF binding sites of MthK WT simulated at 300 mV and different temperature in 1 M KCl. Errors are 95% confidence intervals based on the t-distribution, with the number of observations equal to the number of independent trajectories.

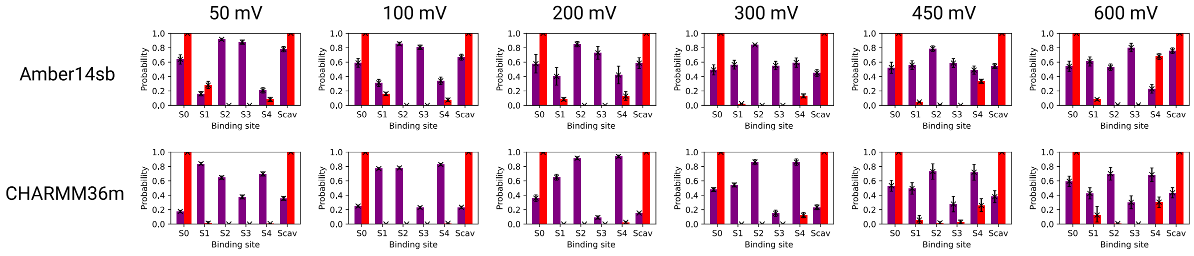

Figure S18: K^+ (purple) and water (red) occupancy in SF binding sites of MthK WT simulated at 323K and different voltages in 1 M KCl. Errors are 95% confidence intervals based on the t-distribution, with the number of observations equal to the number of independent trajectories.

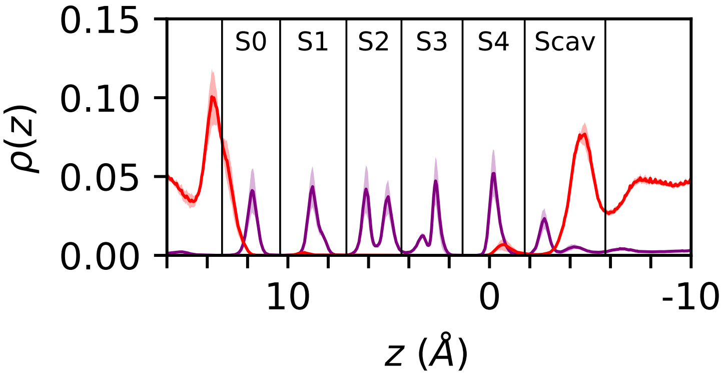

Figure S19: Number density $\rho(z)$ for K^+ (purple) and water (red) along z-axis in MthK WT simulated at 323K and 300 mV in 1 M KCl using Amber14sb. $\int_{z_-}^{z_+} \rho(z)dz$ gives the average number of ions or water molecules between $[z_-, z_+]$ within 4 Å of the pore axis, which is defined by the CoM of C_α atoms of the four T59 and the CoM of C_α atoms of the four G63. Shaded regions represent the standard deviations obtained from 20 independent simulations. A bin size of 0.05 Å was used.

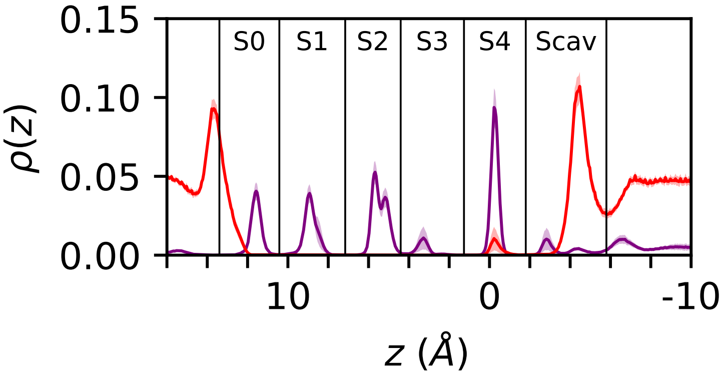

Figure S20: Number density $\rho(z)$ for K^+ (purple) and water (red) along z-axis in MthK WT simulated at 323K and 300 mV in 1 M KCl using CHARMM36m. $\int_{z_-}^{z_+} \rho(z)dz$ gives the average number of ions or water molecules between $[z_-, z_+]$ within 4 Å of the pore axis, which is defined by the CoM of C_α atoms of the four T59 and the CoM of C_α atoms of the four G63. Shaded regions represent the standard deviations obtained from 20 independent simulations. A bin size of 0.05 Å was used.
